# Effects of Dementia-Care Mapping on Residents and Staff of Care Homes: A Pragmatic Cluster-Randomised Controlled Trial

**DOI:** 10.1371/journal.pone.0067325

**Published:** 2013-07-02

**Authors:** Geertje van de Ven, Irena Draskovic, Eddy M. M. Adang, Rogier Donders, Sytse U. Zuidema, Raymond T. C. M. Koopmans, Myrra J. F. J. Vernooij-Dassen

**Affiliations:** 1 Department of Primary and Community Care, Radboud University Nijmegen, Medical Centre, Nijmegen, The Netherlands; 2 Department for Health Evidence, Radboud University Nijmegen, Medical Centre, Nijmegen, The Netherlands; 3 Department of General Practice, University of Groningen, University Medical Center, Groningen, The Netherlands; 4 Scientific Institute for Quality of Healthcare, Radboud University Nijmegen, Medical Centre, Nijmegen, The Netherlands; 5 Kalorama Foundation, Beek-Ubbergen, The Netherlands; 6 Radboud Alzheimer Centre, Radboud University Nijmegen, Medical Centre, Nijmegen, The Netherlands; Federal University of Rio de Janeiro, Brazil

## Abstract

**Background:**

The effectiveness of dementia-care mapping (DCM) for institutionalised people with dementia has been demonstrated in an explanatory cluster-randomised controlled trial (cRCT) with two DCM researchers carrying out the DCM intervention. In order to be able to inform daily practice, we studied DCM effectiveness in a pragmatic cRCT involving a wide range of care homes with trained nursing staff carrying out the intervention.

**Methods:**

Dementia special care units were randomly assigned to DCM or usual care. Nurses from the intervention care homes received DCM training and conducted the 4-months DCM-intervention twice during the study. The primary outcome was agitation, measured with the Cohen-Mansfield agitation inventory (CMAI). The secondary outcomes included residents’ neuropsychiatric symptoms (NPSs) and quality of life, and staff stress and job satisfaction. The nursing staff made all measurements at baseline and two follow-ups at 4-month intervals. We used linear mixed-effect models to test treatment and time effects.

**Results:**

34 units from 11 care homes, including 434 residents and 382 nursing staff members, were randomly assigned. Ten nurses from the intervention units completed the basic and advanced DCM training. Intention-to-treat analysis showed no statistically significant effect on the CMAI (mean difference between groups 2·4, 95% CI −2·7 to 7·6; p = 0·34). More NPSs were reported in the intervention group than in usual care (p = 0·02). Intervention staff reported fewer negative and more positive emotional reactions during work (p = 0·02). There were no other significant effects.

**Conclusions:**

Our pragmatic findings did not confirm the effect on the primary outcome of agitation in the explanatory study. Perhaps the variability of the extent of implementation of DCM may explain the lack of effect.

**Trial Registration:**

Dutch Trials Registry NTR2314.

## Introduction

The prevalence of neuropsychiatric symptoms (NPSs) such as anxiety, apathy, and hallucinations among institutionalised people with dementia is about 80% [1]. These symptoms directly affect the residents’ quality of life and represent a serious challenge to professional caregivers. Staff job dissatisfaction in care homes is frequent and results in high illness absenteeism and turnover rates, which ultimately lead to staff shortages [2]. A strong relationship has been found between high staff turnover and poor resident outcomes such as quality of life. These findings underline the need for interventions to alleviate resident and staff distress [3].

Person-centred care (PCC) is an alternative to conventional task-focused care practices. Evidence suggests that different types of PCC improve both resident and staff outcomes [4]–[6]. Dementia-care mapping (DCM) is a person-centred, multicomponent intervention developed by the Dementia Research Group at Bradford University in the UK and is based on Kitwood’s social-psychological theory of personhood in dementia [5]. This theory states that much of the ill-being that people with dementia experience is due to negative environmental influences, including staff attitudes and care practices.

DCM is a cyclic intervention consisting of three components: systematic observation, feedback to the staff, and action plans. The action plans are based on the observed actual needs of the resident. This method allows for timely initiation of tailor-made interventions at the individual level (residents and caregivers) and the group level (nursing teams), as well as at the levels of multidisciplinary teams, management, and organisations. In short, DCM is a multi-component intervention aiming at synergistically implementing diverse person-centred interventions to improve the quality and effectiveness of care [7].

Chenoweth and colleagues’ cluster-randomised controlled trial (cRCT) [8] compared the effectiveness of PCC training sessions, DCM, and usual, task-focused care. They found that there was less agitation [measured with the Cohen-Mansfield Agitation Inventory (CMAI)] in units providing PCC (mean difference 13·6) and DCM (mean difference 10·9) than in task-focused care. Importantly, this trial demonstrates the effectiveness of DCM in near-ideal conditions. Two researchers carried out the intervention, the setting was well-resourced and tightly controlled, and the care homes were specifically selected for their approaches to care: this renders Chenoweth and colleagues’ study explanatory in character [9]–[11]. Our present study is of a pragmatic nature. Pragmatic studies are intended to maintain the internal validity of RCTs while they are designed and implemented in ways that would better address the demand for evidence about real-world risks. Their purpose is to provide information for making decisions about daily practice. The care homes in this study were not stringently selected so that they would be broadly representative. The nursing staff rather than the researchers were trained to carry out the DCM intervention. This pragmatic cRCT investigated the effectiveness of DCM on resident and staff outcomes.

## Methods

### Participants

The protocol for this trial and supporting CONSORT checklist are available as supporting information; see Checklist S1 and Protocol S1. We recruited care homes via letters of invitation and by approaching care homes that had already had contact with DCM Netherlands. Care for people with dementia in the Netherlands is generally provided in dementia special-care units, where residents generally live in small groups of 5 to 12 people. Staff in Dutch care homes includes nurses, specially trained elderly-care physicians [12], physical therapists, occupational therapists, speech therapists, dieticians, and psychologists, all of whom the care home employs [13]. The study sample consisted of residents with dementia and their formal caregivers. The inclusion criteria for the residents required dementia diagnosed by an elderly-care physician according to the *Diagnostic and statistical manual of mental disorders-IV* criteria for dementia [14], approval of the elderly-care physician for inclusion, age of 65 years or more, at least one NPS, informed consent from the family of the resident, and the ability of the resident to use the common areas, such as the shared living room, for at least 4 h a day. Residents with an estimated life expectancy of 6 weeks or less and those who were physically unable to spend time in common areas of the unit were not included in the study.

We used cluster randomisation to avoid contamination through exchange of information within a care home. We used the minimisation method in the randomisation [15]: we randomised care homes using adaptive weights based on the sizes of the care homes, the sizes of the units (or clusters), and the formal caregiver-to-resident ratios. The study statistician (RD), who was unaware of the identity of the units, used SPSS, version 18 (SPSS, Chicago, Ill.) to randomly allocate them to either the intervention group or the usual care group (allocation ratio 1∶1).

We needed 15 units per arm at baseline to achieve an 80% chance of detecting a true difference of 10·9 for our primary outcome of agitation measured with the CMAI. For this purpose, we also needed an attrition rate, standard deviation, cluster size, and an intraclass correlation coefficient (for patients in a unit) similar to Chenoweth and colleagues, with a maximum correlation of 0·3 between an organisation’s units. We replaced participants lost to follow-up with new participants throughout the study. The details of the methods are reported in the published protocol [16]. The trial is registered with the Dutch Trials Registry, number NTR2314 (http://www.trialregister.nl/trialreg/admin/rctview.asp?TC=2314).

### Ethical Statement

Written informed consent was obtained from the family of the residents. In those cases in which the resident signed the informed consent form, also the family or legal representative provided a signature for consent. The Committee on Research Involving Human Subjects in the Arnhem-Nijmegen Region approved the study participation.

### Procedures

The managers of the units of care homes allocated to the intervention selected staff members who were competent and interested in becoming certified dementia-care mappers. DCM Netherlands provided a guideline specifying the required competences. Ten staff members, two from each intervention care home, attended the basic and advanced training given by DCM Netherlands and became certified dementia-care mappers [16]. Advanced users are able to observe, report, provide feedback to the staff, and instruct and support them in drawing up action plans. After the training, a member of DCM Netherlands and the researchers (AP and GV) gave the intervention care homes a DCM organisational briefing day. After completing the DCM training and attending the organisational briefing day, the trained mappers were to carry out at least two DCM cycles. Each DCM cycle consists of observation, feedback, and action plans.

The control group residents received usual care during the trial. We defined usual care as the continuation of daily care practices without implementation of DCM. The control care homes were offered the DCM training after the trial [16].

### Outcomes

The study outcome measures were assessed at the resident and staff levels. The primary outcome measure at the resident level was agitation, assessed with the CMAI. This assessment instrument consists of 29 items about agitation and aggression and has been validated for use in care homes in the Netherlands [17]. The CMAI measures the frequency (on a seven-point scale from never to several times an hour) of agitation during the preceding 2 weeks (total score range: 29–203). We also assessed NPSs and quality of life as secondary outcome measures. We assessed the NPSs with the Neuropsychiatric Inventory – Nursing Home (NPI-NH) version, a comprehensive assessment scale including the following symptoms: delusions, hallucinations, agitation, depression, anxiety, euphoria, apathy, disinhibition, irritability, aberrant motor behaviour, night-time disturbances, and eating change [18]. The frequency (F) is rated on a four-point (1–4) Likert scale and the severity (S) is rated on and a three-point (1–3) Likert scale, yielding an F times S score. When a symptom is not present, the F and S scores are both zero. The F times S score thus contains information about prevalence, frequency, and severity (range: 0–12 for each symptom). We used the Global Deterioration Scale (GDS) to assess the severity of dementia [19]. The residents’ quality of life was measured with the Qualidem [20] and the EuroQol 5D [21]. The Qualidem includes 37 items and is a multidimensional scale specifically designed for institutionalised residents with dementia. The authors of the Qualidem state that, in case of severe dementia (GDS 7), 18 instead of 37 items can be applied. Therefore, patients in GDS 7 and those in GDS 1–6 are frequently analysed separately [22]. We decided to use only the subscales that were applicable to patients in all stages of dementia. Because not all items were applicable to patients with GDS 7, the maximum score would differ on some subscales for patients in GDS 7 and patients in GDS 1–6. Therefore, we determined the maximum scores for both groups with the applicable items, and converted the original scores into percentages of the maximum score (scale 0–100). This way, we could analyse the data for both groups together. Furthermore, we collected the following demographic data at baseline: age, gender, marital status, and country of birth.

We used the General Health Questionnaire (GHQ)-12 as the primary outcome measure at the staff level to measure stress-related symptoms [23], [24]. We also assessed job experience and job satisfaction as secondary outcome measures using two validated Dutch questionnaires: the Questionnaire about Experience and Assessment of Work (QEAW) [25] and the Maastricht Job Satisfaction Scale for Healthcare (MJSS-HC) [26]. The following staff demographics were collected: age, gender, marital status, country of birth, and previous experience with person-centred care.

All nursing staff of the participating units were asked to fill in questionnaires MJSS-HC, QEAW, and GHQ-12. Any staff member who was primarily responsible for a particular resident was also asked to fill in the resident assessment instruments (CMAI, NPI-NH, Qualidem, EuroQol 5D, and GDS). The staff used an internet application (with a personal user name and password) to fill in these questionnaires. All the variables were measured at baseline (T_0_), after the first DCM cycle (T_1_), and after the second DCM cycle (T_2_) with intervals of 4 months between measurements and a time window of 2 months for completion. The study started in October 2010 and lasted till April 2012.

### Statistical analyses

Analyses were based on the principle of intention-to-treat; all questionnaires were analysed according to their randomised condition. The analyses included all initially and newly included residents and staff members from whom we received at least one completed assessment. The effects were evaluated by means of linear mixed-effect models for longitudinal data, with control variables used in the studywise minimisation procedure [15] as covariates and the unit as a random effect, to correct for dependencies within units. To correct for dependencies caused by repeated measurements, we assumed a heterogeneous structure for the residuals. The following effects were estimated for the outcome variables: the main effect of the intervention, the main effect of time (at three points) and the interaction between the group and time. Two-sided values of p<0·05 were deemed statistically significant. Additionally, we imputed missing data for resident questionnaires that were not completed. Missing data were imputed under the missing-at-random assumption and were based on characteristics extracted from the residential files. Because we did not have any other information about the staff, we did not impute missing data for missing staff questionnaires. We used SPSS, version 18 (SPSS, Chicago, Ill.) for statistical analyses.

## Results

### Participants

**Figure 1:**
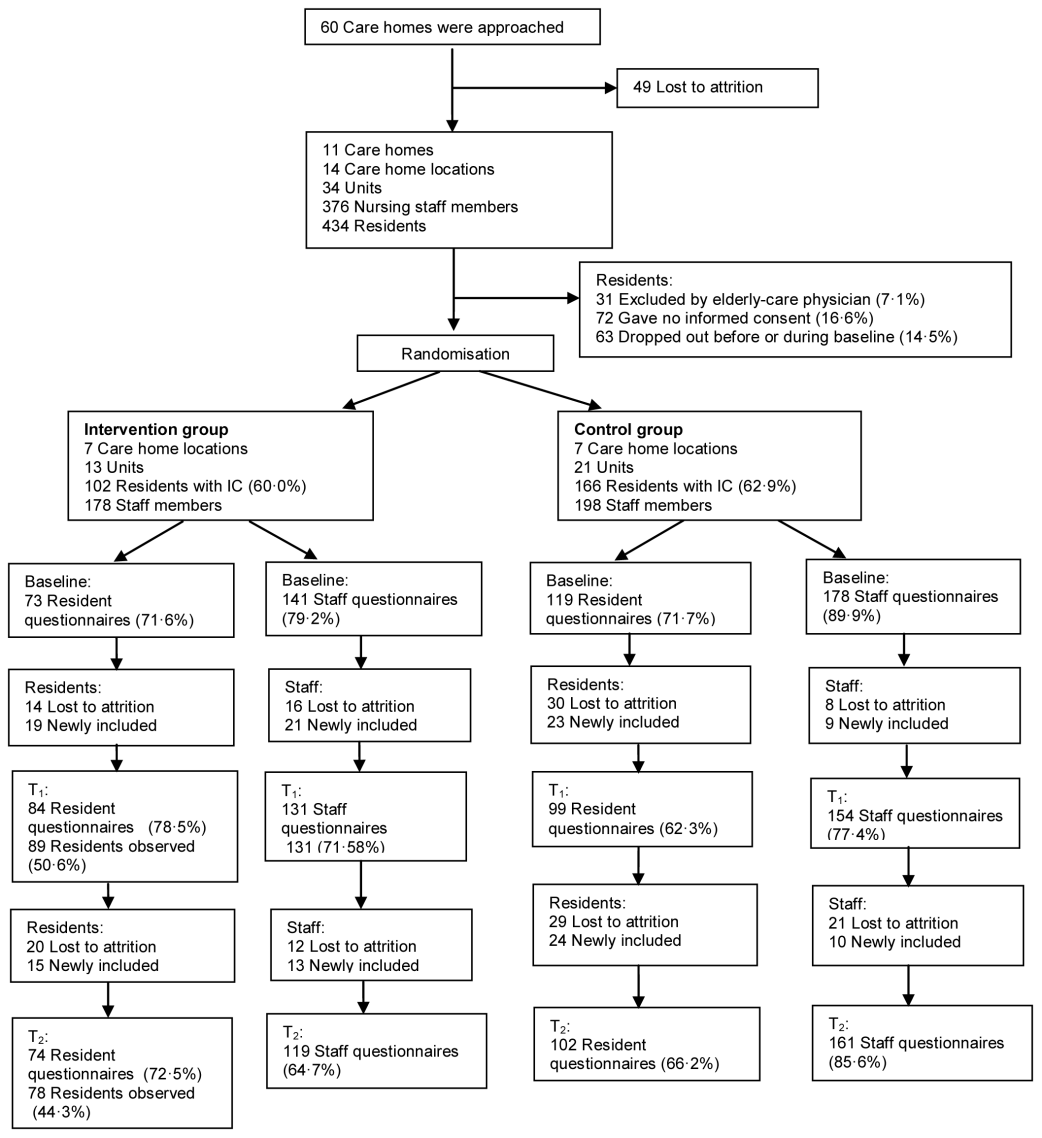
Flowchart detailing numbers of residents and staff.

Across 34 units, 434 residents were eligible (Figure 1). The elderly-care physician excluded 31 (7·1%) of these residents, 72 (16·6%) did not give informed consent, and 63 (14·5%) dropped out before or during the baseline measurement. The 268 (61·8%) residents with informed consent (their own or that of their legal representatives) were included in the study. Ninety-three residents did not complete the study: 87 of them died and 6 moved to another unit or care home. None of the reallocated patients re-entered the study. During the study period, 81 new residents with informed consent were included.

From the same 34 units, 376 nursing staff members were enrolled and 319 (84·8%) of them remained throughout the study. During the study, 53 new staff members were included.

Table 1 shows baseline characteristics of the residents and staff. The mean age of the participating residents was 84·7 (SD 6·3) years and 75·1% were women. Most of the participating staff members were women (98·4%), and their mean age was 43·0 (SD 10·9) years. More than half of them had a previous interest in or experience with person-centred care (56·0% in the intervention group and 55·6% in the control group). The intervention and control groups differed in terms of the proportions of staff in permanent positions. There were no other statistically significant differences in the demographic characteristics at baseline between the intervention and control groups (Table 1).

**Table 1:** Baseline characteristics of residents and staff.

Residents			
	DCM group n = 73	Control group n = 119	p
Mean age in years (SD)	84·6 (6.1)	83·5 (6.6)	0·36
Women	75·0%	73·9%	0·87
Born in the Netherlands	97·5%	97·5%	0·91
**Staff**			
	**DCM group n = 141**	**Control group n = 178**	**p**
Mean age in years (SD)	43·6 (10.4)	42·6 (11·3)	0·32
Women	98·6%	98·3%	0·85
Born in the Netherlands	91·5%	89·9%	0·74
Management position	2·1%	2·2%	0·94
Permanent position	98·5%	91·3%	0·01
Mean years working in the current position (SD)	10·3 (8·3)	10·0 (8·6)	0·45
Mean years working in the organisation (SD)	12·8 (8·1)	10·1 (7·9)	0·43
Mean number of hours a week according to contract (SD)	23·7 (6·7)	22·6 (7·2)	0·92
Previous interest in or experience with person-centred care	56·0%	55·6%	0·94

### Effects of dementia-care mapping on residents and staff

Table 2 shows the results of the primary analysis of the outcome measures. The web appendix provides table S1 and S2, in which the mean scores and standard errors (SE) of all outcome measures can be found.

**Table 2:** Effects of dementia-care mapping on residents and staff based on intention-to-treat analysis.

Residents			
	Baseline (n = 192)	T_1_(n = 182)	T_2_ (n = 175)
	Mean score (SE)	Mean score (SE)	Mean score (SE)
**CMAI: total score p_g_ = 0·340 p_t_ = 0·704 p_gt_ = 0·473**			
DCM	46·61 (1·91)	47·86 (1·88)	48·18 (2·30)
Control group	45·29 (1·56)	44·32 (1·63)	45·81 (1·97)
**NPI-NH: total FxS score p_g_ = 0.226 p_t_ = 0·616 p_gt_ = 0·022**			
DCM	5·35 (0·94)	7·19 (0·95)	6·28 (0·92)
Control group	6·28 (0·88)	4·45 (0·88)	4·13 (0·86)
**Qualidem: total score p_g_ = 0.521 p_t_ = 0.014 p_gt_ = 0.995**			
DCM	64·52 (2·06)	61·88 (2·10)	62·45 (2·19)
Control group	66·31 (1·71)	63·72 (1·81)	64·11 (1·88)
**EuroQol 5D: tariff score p_g_ = 0.158 p_t_ = 0.001 p_gt_ = 0.087**			
DCM	0·39 (0·03)	0·34 (0·03)	0·35 (0·03)
Control group	0·44 (0·02)	0·41 (0·02)	0·36 (0·02)
**Staff**			
	**Baseline (n = 318)**	**T_1_ (n = 284)**	**T_2_ (n = 279)**
	**Mean score (SE)**	**Mean score (SE)**	**Mean score (SE)**
**GHQ-12: total score p_g_ = 0·122 p_t_ = 0·000 p_gt_ = 0·432**			
DCM	17·48(0·33)	15·72 (0·38)	14·57 (0·37)
Control group	16·67 (0·29)	14·89 (0·34)	14·42 (0·32)
**MJSS-HC: total score p_g_ = 0·560 p_t_ = 0·005 p_gt_ = 0·069**			
DCM	76·98 (1·36)	76·40 (1·34)	78·08 (1·40)
Control group	77·29 (1·44)	75·10 (1·43)	75·58 (1·46)
**QEAW: subscale of emotional reactions** **p_g_ = 0·719 p_t_ = 0·000 p_gt_ = 0·015**			
DCM	13·69 (1·51)	23·38 (1·67)	53·28 (1·20)
Control group	9·48 (1·40)	25·97 (1·59)	53·09 (1·12)

SE = standard error.

p_g_ = main effect of the intervention.

p_t_ = main effect of time (at three times).

p_gt_ = interaction between group and time.

CMAI = Cohen-Mansfield agitation inventory.

NPI-NH = Neuropsychiatric Inventory – Nursing Home version.

GHQ-12 = General Health Questionnaire.

MJSS-HC = Maastricht Job Satisfaction Scale for Healthcare.

QEAW = Questionnaire about Experience and Assessment of Work.

We found no significant effect of the DCM intervention on our primary outcome measure, agitation, as measured by the CMAI. The mean difference between groups was 2·4 with a 95% CI of −2·7 to 7·6 and p = 0·34.

There was a significant interaction effect of group and time (p = 0·02) for NPSs in dementia, measured with the NPI-NH. The total F times S score dropped in the control group over time, which means fewer NPSs, but this was not the case in the intervention group. The symptom ‘delusions’ in the NPI-NH also showed a significant interaction effect between time and group; fewer delusions were reported over time in the control group than in the intervention group (p = 0·01).

The quality of life measured with Qualidem showed a significant overall time effect (p = 0·01); poorer quality of life was reported over time in both groups. The subscale ‘social relations’ in the Qualidem showed a significant interaction between group and time (p = 0·03). The score in the control group decreased between baseline and T_1_, while between T_1_ and T_2_, the intervention group showed a decrease in quality of social relations.

Measuring the quality of life in the EuroQol 5D resulted in significantly decreased values, irrespective of the group (p<0·01 for time effect). There were no other statistically significant results at the resident level.

At the staff level, the GHQ-12 showed a significant overall time effect, and fewer stress-related symptoms were reported over time in both groups (p<0·001). There were significant differences between all times: T_1_ compared to baseline (mean difference −1·8, 95% CI −2·3 to −1·2; p<0·001), T_2_ compared to T_1_ (mean difference −0·8, 95% CI −1·4 to −0·2; p = 0·01) and T_2_ compared to baseline (mean difference −2·6, 95% CI −3·2 to −2·0; p<0·001 ). We found no significant intervention effects in the MJSS-HC. The group by time effect in the QEAW was significant for the subscales ‘autonomy’ (p = 0·04) and ‘work pleasure’ (p = 0·03), but these differences were not straightforwardly in favour of the intervention group or the control group. On the subscale ‘emotional reactions’, staff in the intervention group reported significantly fewer negative emotional reactions (such as being hurried or nervous) and more positive emotional reactions (such as being optimistic and relaxed) over time than staff in the control group did (interaction effect p = 0·03). There were no other statistically significant results at the staff level.

In total, 40·9% of all resident questionnaires that should have been filled in by the nursing staff were completely missing (47·6% in the intervention group and 34·6% in the control group). We used multiple imputation in SPSS with the missing-at-random assumption. In this procedure, known relationships that are based on the valid values in the sample, are used to help estimate the missing data. Valid values from the same or from other cases, for example of the CMAI baseline score, unit, or age, were used to create a model for predicting missing values. Analysis with imputed missing data yielded the same results as the linear mixed-effect models analysis. Since there were no differences, we chose to report the findings based on the original data.

## Discussion

The findings of this pragmatic trial did not confirm the effect on the primary outcome of agitation, Chenoweth and colleagues found in their explanatory study [8]. The intervention units reported more NPSs in residents over time than the control group. It could be that, due to the DCM intervention, staff members in the intervention group developed keener observation skills. Additionally, compared to usual care, work-related emotional reactions of the staff developed into more positive ones. This corresponds with the staff outcomes in Jeon and colleagues’ study [27], in which emotional exhaustion scores declined over time in the intervention group but not in the control group. However, considering the sizes of these two effects, their clinical relevance may be limited.

Our lack of evidence for the effect of DCM on agitation seems to contradict the earlier findings of Chenoweth et al. [8]. However, their explanatory trial and our pragmatic trial cannot be compared straightforwardly because of the differences in the study designs [11]. We trained ten nursing staff members from the care homes to perform the DCM intervention without extra support from the research team or DCM Netherlands. This contrasts with Chenoweth’s study [8], in which two research-allied DCM experts performed the DCM intervention in all participating units, thereby minimising the variation of intervention implementation between the units [8], [27], [28]. A Dutch pilot study has found effects of DCM on affective behaviour and verbal agitation. In this study with a before-and-after design, the mappers were from the same highly committed care home [29]. Our results are based on intention-to-treat analysis, which means that all questionnaires were analysed according to their randomised condition, regardless of the actual adherence to the intervention. The variation in adherence across care homes may have masked possible effects of the intervention.

Chenoweth and colleagues [8] selected the care homes for their study because they had task-focused, not person-centred, care systems. In contrast, we used no criteria for the selection of care homes. Indeed, at the start of our study, all care homes claimed to be working with person-centred care systems. It could be that our control group was more like the PCC group than the control group with task-centred care. It is possible that this has attenuated any intervention-induced differences between the intervention and control groups.

The main strengths of this study are the large sample size, and a follow-up of 1 year. We randomised clusters after recruiting the study sample and seeking informed consent from the residents. This way, we controlled for potential selection bias in the control and intervention groups. We used the minimisation method in randomisation to optimise the distribution of baseline characteristics.

This study has several limitations. First, we were unable to blind participating staff to the intervention, given the necessity for staff to be trained in DCM. Second, we cannot guarantee that the units were representative of Dutch care homes – they agreed to participate in this study because they were at least interested in PCC and DCM. While the RCT is the gold standard for testing the effectiveness of an intervention, complex psychosocial interventions such as DCM require process analysis so that we can determine, at least to some extent, the ‘dose-response’ relationship [30].

As already discussed, this trial emulates the real-life situation: the intervention care homes differed in commitment, and nursing staff were trained to map the dementia care. In order to provide information for daily practice, we need to explore the relationship between the extent of the implementation and the effectiveness of DCM.

## Supporting Information

Table S1 **Effects of dementia-care mapping on residents based on intention-to-treat analysis.**
(DOC)

Table S2 **Effects of dementia-care mapping on nursing staff based on intention-to-treat analysis.**
(DOC)

Checklist S1 **CONSORT Checklist.**
(DOC)

Protocol S1 **Trial Protocol.**
(PDF)

Research Proposal S1. (PDF)
