# Supplementary material for: Effects of Dementia-Care Mapping on Residents and Staff of Care Homes: A Pragmatic Cluster-Randomised Controlled Trial
Source: PLoS One. 2013 Jul 2;8(7):e67325. doi: 10.1371/journal.pone.0067325 (PMC3699562; doi:10.1371/journal.pone.0067325)
Supplement: Table S1 — Effects of dementia-care mapping on residents based on intention-to-treat analysis. (DOC) [file pone.0067325.s001.doc]

**Supporting information**

**Table S**1. Effects of dementia-care mapping on residents based on intention-to-treat analysis

|  | **Baseline (n=192)** | **T_1_ (n=182)** | **T_2_  (n=175)** |
| --- | --- | --- | --- |
|  | **Mean score (SE)** | **Mean score (SE)** | **Mean score (SE)** |
| **CMAI: total score p_g_=0·340 p_t_=0·704 p_gt_=0·473** | | | |
| DCM | 46·61 (1·91) | 47·86 (1·88) | 48·18 (2·30) |
| Control group | 45·29 (1·56) | 44·32 (1·63) | 45·81 (1·97) |
| **CMAI: subscale of physically aggressive behaviour p_g_=0·949 p_t_=0·615 p_gt_=0·943** | | | |
| DCM | 11·96 (0·71) | 11·79 (0·64) | 12·00 (0·79) |
| Control group | 12·06 (0·57) | 11·71 (0·55) | 12·14 (0·67) |
| **CMAI: subscale of physically non-aggressive behaviour p_g_=0·480 p_t_=0·413 p_gt_=0·198** | | | |
| DCM | 12·38 (0·86) | 13·62 (0·87) | 13·45 (0·95) |
| Control group | 12·33 (0·71) | 12·05 (0·76) | 12·70 (0·82) |
| **CMAI: subscale of verbally agitated behaviour p_g_=0·138 p_t_=0·068 p_gt_=0·364** | | | |
| DCM | 9·96 (0·77) | 10·11 (0·79) | 9·77 (0·76) |
| Control group | 8·77 (0·64) | 8·89 (0·69) | 7·61 (0·76) |
| **NPI-NH: total severity score (FxS) p_g_=0·226 p_t_=0·616 p_gt_=0·022** | | | |
| DCM | 5·35 (0·94) | 7·19 (0·95) | 6·28 (0·92) |
| Control group | 6·28 (0·88) | 4·45 (0·88) | 4·13 (0·86) |
| **NPI-NH: total workload score p_g_=0·396 p_t_=0·455 p_gt_=0·393** | | | |
| DCM | 2·31 (0·40) | 2·34 (0·38) | 2·33 (0·43) |
| Control group | 2·27 (0·38) | 1·60 (0·36) | 1·78 (0·40) |
| **NPI-NH: severity score (FxS) for the subscale of delusions p_g_=0·143 p_t_=0·618 p_gt_=0·014** | | | |
| DCM | 0·24 (0·12) | 0·60 (0·12) | 0·51 (0·11) |
| Control group | 0·40 (0·11) | 0·22 (0·11) | 0·17 (0·11) |
| **NPI-NH: severity score (FxS) for the subscale of hallucinations p_g_=0·882 p_t_=0·550 p_gt_=0·527** | | | |
| DCM | 0·10 (0·08) | 0·22 (0·11) | 0·19 (0·09) |
| Control group | 0·16 (0·07) | 0·19 (0·10) | 0·12 (0·08) |
| **NPI-NH: severity score (FxS) for the subscale of agitation/agression p_g_=0·862 p_t_=0·501 p_gt_=0·552** | | | |
| DCM | 0·63 (0·17) | 0·62 (0·17) | 0·52 (0·17) |
| Control group | 0·77 (0·16) | 0·49 (0·16) | 0·60 (0·16) |
| **NPI-NH: severity score (FxS) for the subscale of depressed mood p_g_=0·901 p_t_=0·630 p_gt_=0·494** | | | |
| DCM | 0·40 (0·15) | 0·61 (0·17) | 0·40 (0·14) |
| Control group | 0·55 (0·14) | 0·47 (0·15) | 0·45 (0·13) |
| **NPI-NH: severity score (FxS) for the subscale of anxiety p_g_=0·095 p_t_=0·256 p_gt_=0·085** | | | |
| DCM | 0·57 (0·18) | 0·97 (0·20) | 0·93 (0·19) |
| Control group | 0·47 (0·17) | 0·43 (0·18) | 0·35 (0·18) |
| **NPI-NH: severity score (FxS) for the subscale of euphoria p_g_=0·595 p_t_=0·003 p_gt_=0·303** | | | |
| DCM | 0·14 (0·08) | 0·11 (0·06) | 0·06 (0·03) |
| Control group | 0·27 (0·07) | 0·21 (0·05) | 0·02 (0·03) |
| **NPI-NH: severity score (FxS) for the subscale of apathy p_g_=0·579 p_t_=0·853 p_gt_=0·307** | | | |
| DCM | 0·82 (0·27) | 0·97 (0·27) | 0·99 (0·27) |
| Control group | 0·93 (0·25) | 0·70 (0·26) | 0·57 (0·26) |
| **NPI-NH: severity score (FxS) for the subscale of disinhibition p_g_=0·175 p_t_=0·675 p_gt_=0·916** | | | |
| DCM | 0·53 (0·15) | 0·52 (0·15) | 0·48 (0·14) |
| Control group | 0·34 (0·14) | 0·24 (0·14) | 0·21 (0·14) |
| **NPI-NH: severity score (FxS) for the subscale of irritability p_g_=0·537 p_t_=0·450 p_gt_=0·215** | | | |
| DCM | 0·68 (0·16) | 0·81 (0·16) | 0·59 (0·15) |
| Control group | 0·76 (0·14) | 0·49 (0·15) | 0·51 (0·14) |
| **NPI-NH: severity score (FxS) for the subscale of aberrant motor behaviour p_g_=0·149 p_t_=0·119 p_gt_=0·073** | | | |
| DCM | 0·30 (0·12) | 0·80 (0·17) | 0·61 (0·16) |
| Control group | 0·34 (0·11) | 0·26 (0·15) | 0·50 (0·15) |
| **NPI-NH: severity score (FxS) for the subscale of night-time behaviour p_g_=0·870 p_t_=0·458 p_gt_=0·986** | | | |
| DCM | 0·31 (0·12) | 0·43 (0·15) | 0·38 (0·13) |
| Control group | 0·34 (0·11) | 0·47 (0·13) | 0·38 (0·12) |
| **NPI-NH: severity score (FxS) for the subscale of eating change p_g_=0·938 p_t_=0·067 p_gt_=0·069** | | | |
| DCM | 0·58 (0·22) | 0·52 (0·18) | 0·62 (0·18) |
| Control group | 0·99 (0·21) | 0·39 (0·17) | 0·29 (0·17) |
| **Qualidem: total score p_g_=0·521 p_t_=0·014 p_gt_=0·995** | | | |
| DCM | 64·52 (2·06) | 61·88 (2·10) | 62·45 (2·19) |
| Control group | 66·31 (1·71) | 63·72 (1·81) | 64·11 (1·88) |
| **Qualidem: subscale of care relationship p_g_=0·509 p_t_=0·500 p_gt_=0·757** | | | |
| DCM | 68·79 (2·61) | 69·36 (2·35) | 70·87 (2·64) |
| Control group | 70·50 (2·07) | 71·37 (2·00) | 72·07 (2·24) |
| **Qualidem: subscale of positive affect p_g_=0·963 p_t_=0·000 p_gt_=0·292** | | | |
| DCM | 73·15 (3·13) | 67·02 (3·31) | 69·83 (3·40) |
| Control group | 73·57 (2·65) | 68·57 (2·88) | 67·25 (2·94) |
| **Qualidem: subscale of negative affect p_g_=0·385 p_t_=0·274 p_gt_=0·911** | | | |
| DCM | 63·36 (3·03) | 61·10 (3·19) | 61·03 (3·15) |
| Control group | 66·55 (2·64) | 63·97 (2·74) | 65·22 (2·69) |
| **Qualidem: subscale of restless, tense behaviour p_g_=0·167 p_t_=0·468 p_gt_=0·385** | | | |
| DCM | 48·27 (3·82) | 44·04 (3·89) | 47·43 (4·07) |
| Control group | 53·77 (3·17) | 53·81 (3·39) | 53·28 (3·51) |
| **Qualidem: subscale of social relations p_g_=0·819 p_t_=0·036 p_gt_=0·029** | | | |
| DCM | 58·97 (2·96) | 58·70 (2·97) | 54·65 (3·02) |
| Control group | 59·79 (2·46) | 54·05 (2·58) | 55·84 (2·61) |
| **Qualidem: subscale of social isolation p_g_=0·241 p_t_=0·379 p_gt_=0·310** | | | |
| DCM | 66·05 (2·84) | 63·67 (3·04) | 61·77 (3·21) |
| Control group | 68·55 (2·26) | 66·49 (2·61) | 69·63 (2·73) |
| **EuroQol 5D: tariff score p_g_=0·158 p_t_=0·001 p_gt_=0·087** | | | |
| DCM | 0·39 (0·03) | 0·34 (0·03) | 0·35 (0·03) |
| Control group | 0·44 (0·02) | 0·41 (0·02) | 0·36 (0·02) |
| **EuroQol 5D: VAS score p_g_=0·475 p_t_=0·200 p_gt_=0·336** | | | |
| DCM | 57·41 (2·95) | 53·85 (2·98) | 57·58 (3·00) |
| Control group | 59·57 (2·48) | 58·78 (2·62) | 58·73 (2·61) |
| **Severity of dementia: GDS p_g_=0·722 p_t_=0·208 p_gt_=0·945** | | | |
| DCM | 5·46 (0·18) | 5·54 (0·17) | 5·61 (0·17) |
| Control group | 5·37 (0·15) | 5·44 (0·15) | 5·56 (0·14) |

SE= standard error

p_g_ = main effect of the intervention

p_t_ = main effect of time (at three times)

p_gt_ = interaction between group and time

CMAI= Cohen-Mansfield agitation inventory

NPI-NH= Neuropsychiatric Inventory – Nursing Home version

VAS= Visual Analogue Scale

GDS= Global Deterioration Scale
