# Supplementary material for: Effects of Dementia-Care Mapping on Residents and Staff of Care Homes: A Pragmatic Cluster-Randomised Controlled Trial
Source: PLoS One. 2013 Jul 2;8(7):e67325. doi: 10.1371/journal.pone.0067325 (PMC3699562; doi:10.1371/journal.pone.0067325)
Supplement: Table S2 — Effects of dementia-care mapping on nursing staff based on intention-to-treat analysis. (DOC) [file pone.0067325.s002.doc]

**Table S2. Effects of dementia-care mapping on nursing staff based on intention-to-treat analysis**

|  | **Baseline (n=318)** | **T_1_ (n=284)** | **T_2_ (n=279)** |
| --- | --- | --- | --- |
|  | **Mean score(SE)** | **Mean score(SE)** | **Mean score(SE)** |
| **GHQ-12: total score p_g_= 0·122 p_t_=0·000 p_gt_=0·432** | | | |
| DCM | 17·48 (0·33) | 15·72 (0·38) | 14·57 (0·37) |
| Control group | 16·67 (0·29) | 14·89 (0·34) | 14·42 (0·32) |
| **MJSS-HC: total score p_g_= 0·560 p_t_= 0·005 p_gt_=0·069** | | | |
| DCM | 76·98 (1·36) | 76·40 (1·34) | 78·08 (1·40) |
| Control group | 77·29 (1·44) | 75·10 (1·43) | 75·58 (1·46) |
| **MJSS-HC: subscale of satisfaction with the manager**  **p_g_= 0·083 p_t_= 0·925 p_gt_=0·072** | | | |
| DCM | 11·09 (0·39) | 11·32 (0·39) | 11·34 (0·40) |
| Control group | 10·42 (0·42) | 10·13 (0·42) | 10·06 (0·43) |
| **MJSS-HC: subscale of promotion p_g_= 0· 644 p_t_= 0·039 p_gt_=0·051** | | | |
| DCM | 9·95 (0·25) | 9·80 (0·26) | 10·21 (0·27) |
| Control group | 10·08 (0·26) | 9·70 (0·27) | 9·67 (0·27) |
| **MJSS-HC: subscale of quality of care p_g_= 0·654 p_t_= 0·000 p_gt_=0·069** | | | |
| DCM | 9·50 (0·34) | 9·19 (0·34) | 9·88 (0·35) |
| Control group | 10·07 (0·37) | 9·43 (0·37) | 9·76 (0·37) |
| **MJSS-HC: subscale of opportunity to grow p_g_= 0·911 p_t_= 0·503 p_gt_=0·108** | | | |
| DCM | 10·90 (0·24) | 10·83 (0·23) | 11·15 (0·24) |
| Control group | 11·05 (0·24) | 10·91 (0·24) | 10·81 (0·24) |
| **MJSS-HC: subscale of contact with colleagues p_g_= 0·943 p_t_= 0·087 p_gt_=0·675** | | | |
| DCM | 12·15 (0·19) | 12·04 (0·20) | 12·15 (0·21) |
| Control group | 12·25 (0·20) | 11·98 (0·20) | 12·16 (0·21) |
| **MJSS-HC: subscale of contact with patients p_g_= 0·674 p_t_= 0·402 p_gt_=0·567** | | | |
| DCM | 12·17 (0·14) | 12·14 (0·14) | 12·21 (0·15) |
| Control group | 12·36 (0·13) | 12·14 (0·14) | 12·23 (0·14) |
| **MJSS-HC: subscale of clarity of task p_g_= 0·725 p_t_= 0·066 p_gt_=0·806** | | | |
| DCM | 11·10 (0·19) | 10·94 (0·19) | 11·02 (0·20) |
| Control group | 11·08 (0·20) | 10·81 (0·20) | 10·89 (0·20) |
| **QEAW: subscale of autonomy**  **p_g_= 0·387 p_t_= 0·009 p_gt_=0·037** | | | |
| DCM | 44·37 (1·60) | 45·89 (1·55) | 43·86 (1·59) |
| Control group | 40·59 (1·55) | 43·41 (1·52) | 44·51 (1·53) |
| **QEAW: subscale of problems with task**  **p_g_= 0·881 p_t_= 0·001 p_gt_=0·770** | | | |
| DCM | 17·83 (1·32) | 19·90 (1·33) | 19·55 (1·38) |
| Control group | 17·84 (1·33) | 19·87 (1·35) | 20·40 (1·36) |
| **QEAW: subscale of work pleasure p_g_= 0·335 p_t_= 0·343 p_gt_=0·030** | | | |
| DCM | 11·05 (1·98) | 9·69 (2·00) | 10·28 (2·02) |
| Control group | 7·26 (2·03) | 9·30 (2·05) | 6·22 (2·05) |
| **QEAW: subscale of job change p_g_= 0·603 p_t_= 0·092 p_gt_=0·703** | | | |
| DCM | 16·51 (3·17) | 18·27 (3·25) | 16·58 (3·34) |
| Control group | 17·33 (3·15) | 21·37 (3·24) | 19·64 (3·25) |
| **QEAW: subscale of emotional reactions** **p_g_= 0·719 p_t_= 0·000 p_gt_=0·015** | | | |
| DCM | 13·69 (1·51) | 23·38 (1·67) | 53·28 (1·20) |
| Control group | 9·48 (1·40) | 25·97 (1·59) | 53·09 (1·12) |

SE= standard error

p_g_ = main effect of the intervention

p_t_ = main effect of time (at three times)

p_gt_ = interaction between group and time

GHQ-12= General Health Questionnaire

MJSS-HC= Maastricht Job Satisfaction Scale for Healthcare

QEAW= Questionnaire about Experience and Assessment of Work
