# Supplementary material for: Effects of Dementia-Care Mapping on Residents and Staff of Care Homes: A Pragmatic Cluster-Randomised Controlled Trial
Source: PLoS One. 2013 Jul 2;8(7):e67325. doi: 10.1371/journal.pone.0067325 (PMC3699562; doi:10.1371/journal.pone.0067325)
Supplement: Research Proposal S1. — (PDF) [file pone.0067325.s005.pdf]

Algemene gegevens / General Information

Programma / Programme	:	
Subsidieronde / Subsidy round	:	**Ronde 10 - grand application E&K**
Projecttitel / Project title	:	**An RCT study of (cost)effectiveness of Dementia Care Mapping intervention in nursing home settings**
Projecttaal / Project language	:	**Engels / English**
Geplande startdatum / Planned start date	:	**01-10-2009**
Geplande duur / Planned duration	:	**36 maanden / months**
Datum indienen / Date of application	:	**30-08-2009**
Projecttype / Project type	:	**Implementatieproject / Implementation project**
Vervolg eerder ZonMw-project / Continuation previously funded project ZonMw	:	**Nee / No**

Aanvrager / Applicant

Dr. I Draskovic
T: 0243619641 | *F:* | *E:* i.draskovic@kwazo.umcn.nl

Universitair Medisch Centrum St. Radboud
Biomedische Wetenschappen en Extramurale Geneeskunde
Kwaliteit van Zorg, 117 KWAZO

Postbus 9101
6500 HB NIJMEGEN

Projectleden / Project members

Prof. dr. T. van Achterberg (Mede aanvrager)
Functie / Position: Professor Nursing Sciences | *Opleiding / Education:*
Studierichting / Subject:
T: 0243619529 | *F:* | *E:*

Universitair Medisch Centrum St. Radboud
IQ healthcare

Postbus 9101
6500 HB NIJMEGEN

Dr. EMM Adang (Mede aanvrager)
Functie / Position: Associate professor | *Opleiding / Education:*
Studierichting / Subject:
T: 0243615300 | *F:* | *E:*

Radboud University Nijmegen Medical Centre
Department of Epidemiology, Biostatistics and HTA

Postbus 9101
6500 HB NIJMEGEN

Prof. dr. D Brooker (Mede aanvrager)
Functie / Position: Professor of Dementia Care Practice and Research | *Opleiding / Education:*
Studierichting / Subject:
T: +44 (0) 1274 235726 | *F:* | *E:*

University of Bradford
School of Health Studies
Bradford Dementia Group

Unity Building, Trinity Road 25
BD5 0BB Bradford

R Donders (Mede aanvrager)
Functie / Position: Senior researcher | *Opleiding / Education:*
Studierichting / Subject:
T: 0243617794 | *F:* | *E:*

Radboud University Nijmegen Medical Centre
Department of Epidemiology, Biostatistics and HTA

Postbus 9101
6500 HB NIJMEGEN

Dr. I Draskovic (Projectleider en penvoerder)
Functie / Position: Senior researcher | *Opleiding / Education:*
Studierichting / Subject:
T: 0243619641 | *F:* | *E:* I.Draskovic@iq.umcn.nl

Universitair Medisch Centrum St. Radboud
Scientific Institute for Quality of Healthcare

Postbus 9101
6500 HB NIJMEGEN

Prof. dr. RTCM Koopmans (Mede aanvrager)
Functie / Position: Professor Nursing Home Medicine | *Opleiding / Education:*
Studierichting / Subject:
T: 0243614036 | *F:* | *E:*

Radboud University Nijmegen Medical Centre
Primary Care Medicine
Centre for Primary Care Medicine, Elderly Medicine and Public Health

Geert Grooteplein-Noord 21
6525 EZ NIJMEGEN

Drs. EARJ Lohman (Bestuurlijk verantwoordelijke)
Functie / Position: chairman Hospital Board | *Opleiding / Education:*
Studierichting / Subject:
T: 024-3619603 | *F:* 024-3619162 | *E:*

Universitair Medisch Centrum St. Radboud
Raad van Bestuur

Postbus 9101
6500 HB NIJMEGEN

Mw. A Post (Projectadviseur)
Functie / Position: Project Manager Dementia Care Mapping | *Opleiding / Education:*
Studierichting / Subject:
T: 0511467178 | *F:* | *E:*

Zorgkwadrant Fryland Oost
locatie Berchhiem

Postbus 76
9250 AB BURGUM

Prof. dr. MJFJ Vernooij-Dassen (Mede projectleider)
Functie / Position: Professor Psychosocial aspects of dementia care | *Opleiding / Education:*
Studierichting / Subject:
T: 024 3616643 | *F:* | *E:*

Universitair Medisch Centrum St. Radboud
Verpleeghuisgeneeskunde
117 VPHG

Postbus 9101
6500 HB NIJMEGEN

Dr. S Zuidema (Mede aanvrager)
Functie / Position: Nursing Home Physician | *Opleiding / Education:*
Studierichting / Subject:
T: 0243618834 | *F:* | *E:*

Radboud University Nijmegen Medical Centre
Primary Care Medicine
Centre for Primary Care Medicine, Elderly Medicine and Public Health

Geert Grooteplein-Noord 21

6525 EZ NIJMEGEN

Projectgegevens / Project information

Aandachtsgebieden / Focus

Optimalisatie van verpleegkundig handelen

- Vroegsignalering
- Zorgplannen

Samenvatting / Summary

OBJECTIVES/RESEARCH QUESTIONS
Effectiveness and efficiency of nursing home dementia care is sub-optimal with high levels of resident neuropsychiatric symptoms and staff work stress. (1-7) The main objective of the present study is to evaluate (cost)effectiveness of Dementia Care Mapping (DCM) in nursing home dementia care by performing a large scale RCT study. DCM is a method by which person centred care (8,9) can be integrated in all facets of nursing home dementia care. (10-12) There is a strong evidence that person centred care may alleviate both the resident and the staff problems. (13-18)

Research question
To what extent is Dementia Care Mapping intervention effective in reducing neuropsychiatric symptoms (PRIMARY), staff distress (SECONDARY), and costs of dementia care on Psycho-geriatric units (PGUs) (SECONDARY)?

STUDY DESIGN
Cluster RCT design with PGUs as clusters and sequential balancing minimisation as allocation method. PGUs in the control group will receive the usual care.

STUDY POPULATION(S)/datasets:
280 nursing home residents with dementia and one or more neuropsychiatric symptoms, and their professional caregivers from 18-20 PGUs in the Netherlands.

INTERVENTION:
1. Dementia Care Mapping (DCM) Organisational Briefing Day
2. Staff trainings (Certified DCM User)
3. Three DCM-cycles (single cycle: observation, feed-back, development and implementation of action plan(s), evaluation actions).

I. Residents:
1. Neuropsychiatric symptoms - primary (CMAI, NPI-NH), quality of life (WIB, DQol)

II. Staff:
1. Work-stress – secondary (GHQ); 2.Stress related absenteeism, and turnover -secondary; 3. Job satisfaction (MAS en VAS) - secondary.

SAMPLE SIZE CALCULATION/DATA ANALYSIS
- power- 80%
- effect size 10.90 (SD=3.8) on CMAI(10),
- ICC - 0.05,
- cluster size - 14 observations,
- alpha - .05,
- 20% attrition rate per 8 months
- sample size - N=260-280 or 18-20 PGUs.

The effects on the primary outcomes will be evaluated using linear mixed effect model.

ECONOMIC EVALUATION
The economic evaluation is based on the general principles of cost-effectiveness analysis from a societal viewpoint.

TIME SCHEDULE
Months 1-12
1. Symposium, workshops for the nursing home staff and managers, and professional organizations;
2. Recruitment and selection of nursing homes, randomisation of eligible SCUs;
3. DCM staff training

Months 13 - 24
1. Baseline measurement
2. 4 months DCM-cycle
3. T-1 Measurement
4. 4 months DCM-cycle

5. T-2 Measurement

Months 25-36
1. Analyses, dissemination
2. Closing symposium

Samenvatting
Effectiviteit en efficiëntie van dementiezorg in verpleeg- en verzorgingshuizen (VVH) is suboptimaal met een hoge prevalentie van neuropsychiatrische symptomen bij cliënten en veel werkstress bij de staf. (1-7) Ons doel is om de effectiviteit en efficiency van Dementia Care Mapping (DCM) interventie in VVH dementiezorg te evalueren door middel van een RCT studie. DCM is een methode om persoonsgerichte zorg (8,9) te integreren in VVH-dementiezorg. (10-12) Onderzoek laat zien dat persoonsgerichte zorg de problemen van mensen met dementie en hun verzorgers kan verhelpen. (13-18)

Onderzoeksvraag
In hoeverre is DCM-interventie effectief in het verminderen van prevalentie en intensiteit van neuropsychiatrische symptomen (PRIMAIR), werk stress (SECUNDAIR) en kosten van dementiezorg in verpleeg- en verzorgingshuizen (SECUNDAIR)?

STUDIE DESIGN
Cluster RCT design met psychogeriatrische afdelingen (PGAs) als clusters and using ‘sequential balancing’ minimisatie methode voor de verdeling van PGAs. Controle groep krijgt gebruikelijke zorg.

STUDY POPULATION(S)/datasets:
280 verpleeg- en verzorgingshuisbewoners met dementie en een of meer neuropsychiatrische symptomen; hun professionele verplegers/verzorgers

INTERVENTIE:
1. Dementia Care Mapping (DCM) Briefing Dag voor de organisaties.
2. Staff training – Certified Users.
3. Drie DCM-cycli (1 cyclus: observatie, feed-back, ontwikkeling/implementatie actie plannen, evaluatie).

UITKOMSTEN
Cliënten met dementie:
1. Neuropsychiatrische symptomen - primair (CMAI, NPI), kwaliteit van leven (WIB, DQol) -sec.

Staf:
1. Werkstress – secundair (GHQ); 2. Stress - gerelateerde ziekteverzuim, en personeelsverloop -secundair; 3. Werktevredenheid (MAS en VAS) - secundair

POWER ANALYSE/DATA ANALYSIS
- power - 80%,
- effect 10.90 (SD=3.8)CMAI,
- ICC van 0.05,
- 14 observaties per afdeling,
- alpha = .05
- drop-out = 20%,
- steekproefgrote N=260-280 of 18-20 PGUs.

ECONOMISCHE EVALUATIE
De economische evaluatie is gebaseerd op de algemene principes van kosteneffectiviteitanalyse vanuit maatschappelijk perspectief.

TIJDSPLANNING
Maanden 1-12
1. Symposium (info, werving)
2. Werving, selectie, randomisatie PGUS
3. DCM training

Maanden 13 - 24
1. DCM-cycli

Maanden 25-36
1. Analyses and reportage;
2. Slotsymposium

Trefwoorden / Keywords
Alzheimer's_disease; Dementia; Dementia_care; Dementia_Care_Mapping; Nursing_homes; Neuro-psychiatric_symptoms; Person_centred_care

Samenwerking / Collaboration

Samenwerking tussen onderzoek en praktijk / Cooperation between research and practice:
Ja / Yes

Organisaties

Stichting Kalorama
Nieuwe Holleweg 12
6573 DX BEEK UBBERGEN

University of Bradford
School of Health Studies
Bradford Dementia Group
Unity Building, Trinity Road 25
BD5 0BB BRADFORD

Zorgkwadrant
De Friese Wouden
DCM Nederland
Prinses Margrietstraat 1
9251 HA BURGUM

Inhoud / Content

Probleemstelling / Problem definition
A. HEALTHCARE PROBLEM:
Prevalence of neuropsychiatric symptoms among nursing home residents with dementia is 83%. (3,19-21) In addition to directly affecting the residents' quality of life, these symptoms represent a serious challenge for the professional caregivers. (13,22) Staff job dissatisfaction brings about high illness absenteeism (5.4%) and turnover rates (brt. 14,1%, net. 6.3%) (23), ultimately leading to staff shortage (41% difficult to fill job openings in 2007). (6, 23-28) A strong relationship found between high employee turnover and poor resident outcomes (6,13, 14) suggests that present effort put into dementia care does not result in sufficient quality and (costeffectiveness of care. In order to be able to provide optimal dementia care, staff often needs additional training (4,26,29,30). Except for DCM, no other intervention offers an integral person centred approach to dementia care. Other interventions based on person centred care, such as snoezelen (14,15) and person-centred bathing (31,32) have a more limited scope (residents or staff alone, single psychosocial intervention, or a single caregiving situation). In addition, these interventions often do not include systematic adaptations in management style and organizational climate. It can be expected that all these conditions need to operate synergistically if we are to sustainably improve effectiveness, efficiency and quality of dementia care in nursing homes. We will describe how DCM- intervention fulfils these criteria in the 'Strategy: Intervention' section below. What we hope to achieve is 1. reduction in frequency and intensity of neuropsychiatric symptoms (CMAI, NPI-NH; primary outcome); 2. improvement in quality of life of dementia patients (DCM- WIB, DQoL); 3. improvement in staff-resident interactions, and in staff job satisfaction (DCM: PE/PD, MAS, VAS); 4. reduction in job related stress, absence and stuff turnover (GHQ, organisational data bases). In addition we will perform a cost effectiveness analysis in order to find out whether our intervention positively affects the costs of care. Psychometric characteristic of all instruments will be discussed in the section Strategy (Plan van aanpak).
B. THE DISEASE / CONDITION
Dementia (DSM IV-TR criteria).

C. THE (SUB-)GROUP OF PATIENTS
The subgroup of 'patients' targeted in this proposal consists of nursing home residents diagnosed with dementia (DSM IV-TR criteria) and suffering from one or more neuropsychiatric symptoms including agitation, depression, aggression and apathy.

D. INDICATE HOW VARIATIONS IN SEX, AGE OR CULTURAL BACKGROUND ARE TAKEN INTO ACCOUNT
Demographic characteristics of the participants, such as gender, socio-economic status (SES) and land of origin will be registered and included into analysis in order to control for confounding.

E. DESCRIBE THE USUAL CARE IN THE NETHERLANDS FOR THE SUBGROUP OF PATIENTS INVOLVED
Several governmental, professional and scientific reports assess present nursing home care as ineffective and inefficient due to high levels of patient as well as staff distress, and high absenteeism and turnover rates (23, 26, 27, 29, 33). In the past year, of the 229 inspected nursing homes (NHs) with a psychogeriatric unit, 32 (14%) have received a warning by the Health Inspectorate (HI). (5, 7) Presently, task –centred care is still more prevalent than person centred care. (13, 22) The 'Visible Care' report (7) stresses the need for urgen action regarding the following outcomes: fall and medication incidents, incontinence, and problem behaviour (in dementia: neuro-psychiatric symptoms). The new guidelines on problem behaviour (18) stress the need to prevent excessive use of anti-psychotics. Professional organizations (NVVA, V&VN) endorse and recommend the use of the principles of person centred care. However, what is missing is a systematic method to implement the principles in all facets of nursing home dementia care. While guidelines and protocols are necessary, they are too abstract to serve the purpose of staff development and application of person centred care in daily practice.(17, 18) DCM-intervention offers

a set of methods to these aims.

F. WHO ARE INVOLVED IN THE USUAL CARE AND WHAT THEIR PARTICIPATION IN THIS PROPOSAL IS?
Nursing staff, psychologists, nursing home physicians, physical therapists, occupational therapists. Representatives of these groups will be part of our (broader) research group.

G. MOTIVATION FOR THE (EFFECTIVENESS OF) THE INTERVENTION:
Person-centred care has been praised as important for the well-being of residents with dementia (15, 16, 22, 34-36). Only recently, evidence is accruing about its relation to the well-being of the staff (8, 9, 14). Dementia Care Mapping method is successful in alleviating problems on both sides. Duch pilot study confirmed positive effect for both the residents and the staff. (37)

h. Patient compliance
Not applicable

Relevantie / Relevance

A. MOTIVATION FOR THE CHOSEN THEME:
The theme of the present proposal is Optimisation of nursing care with focus on early signalling and care planning. Implementation of the principles of person centred care by means of the DCM-intervention insures closer contact and better interaction between the residents and the staff. This way, the staff are able to signal problem behaviours before they escalate. DCM-intervention provides training on how to deal with these problems. Regarding the care planning focus, DCM-intervention provides training in developing and implementing care plans and actions that will improve the quality of care on a long term basis. (11, 35, 38)

B. CONTRIBUTION OF THE RESULTS TO THE RESOLUTION OF THE HEALTHCARE PROBLEM:
Prevalence of neuropsychiatric symptoms in nursing home dementia patients is very high (3, 21) and it poses a serious problem for the residents. At the same time, European report on work-stress established that, for the seven countries included in the study (Denmark, Finland, France, Germany, the Netherlands, Spain, Sweden), 'health and social services' is one of the 2 sectors identified as being most at risk. (39, 40) In addition, the most recent survey by the professional organisation for the nursing staff (VENVN) shows that more that 80% of the nursing staff experiences work as ‘difficult’, 35% of these respondents has difficulty with work stress while 40% has difficulty with both the stress and the physical aspects of care (41). Furthermore, 1/3 of the nursing staff is confronted with aggressive behaviour on daily or weekly basis. (42) The results from the present study will inform practice of nursing home care regarding the effectiveness of Dementia Care Mapping intervention in alleviating the present problems experienced by the residents, the nursing staff and the Psycho-geriatric Units as a whole. This may facilitate broad implementation of this method.

C. ARE THERE ANY STUDIES UNDERWAY SIMILAR TO THE PRESENT STUDY PROPOSAL OR RELATED TO THE HEALTHCARE PROBLEM OF THE PRESENT STUDY PROPOSAL (NATIONAL OR INTERNATIONAL):
There is an Australian RCT study on effects of DCM on service delivery, care planning, care environment and the resident’s quality of life/well-being in progress. (10) However we do not know of any cost-effectiveness RCT study, such as ours, in progress.

D. ARE THERE ANY RECENT REPORTS BY NATIONAL ADVISORY BOARDS ON THE SUBJECT OF YOUR PROPOSAL?
The need to improve nursing home (dementia) care has been acknowledged in several
(government) reports (4, 5, 7, 29) and by Professional organizations (23, 41). Dementia report of the Health Council of the Netherlands (29) states that “Quality and organization of care for dementia patients must be improved. The training of professional caregivers and the quality of home care and institutional care is characterized by major variations. Consensus is lacking on an integrated package of care services for dementia patients.” Recent reports on high levels of aggressive confrontations in nursing homes (5, 7) are just the most extreme manifestation of the underlying problems in patient-staff interactions which need to be addressed.

E. WHAT IS THE INCIDENCE / PREVALENCE IN THE TARGETED (SUB-) POPULATION

PREVALENCE DEMENTIA IN NH
Currently, in Dutch Nursing Homes a total number of 30.318 demented persons are institutionalised. The prevalence of dementia in the population of nursing home residents is =53% (26-28)

PREVALENCE NEUROPSYCHIATRIC SYMPTOMS IN NH DEMENTIA PATIENTS
In the Netherland, Neuropsychiatric symptoms were observed in more than 80% of the nursing home residents with dementia.(3, 43)

F. ESTIMATE THE POTENTIAL EFFECTS ON HEALTH COMPARED TO USUAL CARE:
Empirical findings from pilot studies suggest sizable effects on the occurrence of neuropsychiatric symptoms (agitation) in nursing home dementia residents (effect size of 11.94, SD=23) obtained with CMAI. (37) In addition, similar effects can be expected with staff job satisfaction (improvement) and work-stress (decrease). (10, 14-16, 37)

G. ESTIMATE THE POTENTIAL EFFECTS ON COSTS FROM THE INTERVENTION(S):

The ratio behind the expected effects of our intervention on costs is the following:
1. Reduced prevalence and intensity of neuropsychiatric symptoms will have positive effects on formal caregivers by reducing their work- stress and by increasing their feeling of competence and job satisfaction.
2. This will have positive effect on productivity and efficiency of care through a reduction in job related illness, absence and turnover, and through more efficiently organized work-processes.
3. Reduced residents' care needs and negative outcomes (e.g., use of psychoactive medication, acute hospitalization or transfer to psychiatric hospitals), may directly reduce the costs of care. (3, 4, 27)

Kennisoverdracht, implementatie, bestendiging / Knowledge transfer, Implementation Consolidation

The target groups are persons with dementia residing in nursing homes, their formal caregivers (professional health care workers) and caregiver organizations, Alzheimer Societies, researchers in dementia care, and policy makers. The final aim is to incorporate the DCM-intervention into the usual care provided by nursing home psycho-geriatric units, since this is a potentially feasible and effective way to enhance the quality of life of patients and improve competences and job satisfaction of the formal caregivers. In addition, the intervention is likely to save costs by reducing care needs and staff absenteeism and turnover rates. In order to support future implementation on a broader scale, the three Alzheimer Centres (Nijmegen, Amsterdam, Limburg) will advocate the importance of increased attention on the interactions between the patient neuropsychiatric symptoms and caregiver competences and (in case of positive outcomes of this study) will urge to implement the DCM programme in their health care regions. An important effort towards broader implementation of the intervention is to train the professionals included in the control group after the study. Furthermore, on a national level, nursing homes will be stimulated to include this intervention into the usual care through the DCM-NL-group and professional organizations (V&VN, LEVV). The dissemination of the findings will be realized through several scientific and professional channels. Through international collaboration with Bradford Dementia Group, University of Bradford, School of Health Studies, UK, the results of the intervention can be compared with previous studies and disseminated. Information about the intervention will be disseminated by publications and presentations both at scientific and professional conferences and it will be made available directly to the participating nursing homes and professional organizations. Furthermore, currently the possibilities are being explored of implementing DCM-training in hospital dementia care (35), and in care for the community dwelling people with dementia involving day care staff, and informal caregivers (DCM-NL group). Finally, problem behaviour is not uncommon in somatic care units in nursing homes. There is no á priori reason why DCM could not be implemented in these settings as well.

Doelstelling / Objective

The main objective of the present study is to evaluate (cost)effectiveness of Dementia Care Mapping (DCM)intervention in nursing home dementia care by performing a large scale RCT study.

Research questions:
1. To what extent is the DCM-intervention effective in reducing dementia residents neuropsychiatric symptoms (primary), improving dementia residents quality of life (secondary), improving staff competencies and job satisfaction (secondary)?

2. To what extent is the DCM-intervention efficient (cost-effective) through a reduction in patient care needs and negative outcomes, reduction in staff stress, absence and turnover rates, and improvement in efficiency of care delivery processes?

Practice goals
To train approximately 20 certified DCM users (the exact number depends on the needs of the control group) which will be an asset for the participating nursing homes.

Plan van Aanpak / Strategy

1. CLINICAL STUDY
A. PRELIMINARY STUDIES BY APPLICANTS ON THE SUBJECT OF THIS PROPOSAL:
Our partner the Friese Wouden initiated a preliminary study on the effectiveness of dementia care mapping. The study was performed by the researchers from the University of Groningen with whom we collaborate within DCM-NL. In this first Dutch pilot study (N=48) positive effects were obtained for patient well being (WIB-scores), anxiety (GIP), agitation (CMAI), positive staff-resident interactions (PEs), and staff satisfaction with contacts with clients (MAS, VAS). (37) The collaborating Bradford dementia group has documented all international studies on the effects of Dementia Care Mapping. The results are summarized in a review by our collaborator prof. D. Brooker and will be described further on in this section. Our co-applicant Prof. Dawn Brooker has performed several studies on the subject of this proposal. (11, 12, 34, 35, 38, 44)

B. DESIGN:
We will evaluate DCM-intervention in Dutch nursing homes using cluster RCT design with Psycho-geriatric Units (PGUs) as clusters. Control group will receive usual care. PGUs in the control group will be placed on the wait list and will receive the intervention after the measurements are completed. We will use a sequential balancing minimisation method (45) in allocating cluster units (PGUs) to either the treatment or the control group. In this method, each factor is dealt with sequentially and when a new cluster (or participant) enters the trial, it is allocated the treatment that leads to improved balance of the first factor over the treatments. If the balance of the first factor was already satisfactory, then the treatment is allocated that leads to improved balance of the second factor and so on. Quantitative methods will be used to study effectiveness and efficiency, and qualitative methods will be used to study facilitators and barriers for a broader implementation of DCM in daily practice.

C. STUDY POPULATION/DATA SOURCES/SEARCH STRATEGY:
Study population consists of 280 patients from nursing home PGUs who are suffering from neuropsychiatric symptoms (CMAI, NPI-NH), and their formal caregivers. We will strive to cover several different regions in the Netherlands. Recruitment of nursing homes and their PGUs will be performed in several ways: e.g., advertising on the DCM-NL website (http://dcmnederland.nl/),

VENVN website (professional organisation for the nursing personnel), and invitational letters to nursing homes.

INCLUSION CRITERIA PATIENTS (residents)
- 65-up years old;
- diagnosed with dementia by a (psycho)geriatrician, neurologist or old age psychiatrist/psychologist;
- who have received an assent by the nursing home physician
- who display at least one neuropsychiatric symptom (CMAI, NPI-NH) such as agitation/aggression, apathy, verbal and physical aggression, severe vocal and physical perseveration, significant wandering, and self-harm;
- who have either provided informed consent themselves, or by their family/ legal guardian

EXCLUSION CRITERIA
- Residents who are in a terminal stage of the illness (estimated life expectancy 6 weeks), or those who are too physically unwell to spend time in common areas of the facility, will not be included in the study.

STOP CRITERIA
- revoking of consent for any reason;
- life threatening disease or severe exacerbation of symptoms

D. INTERVENTION
Dementia Care Mapping method has been developed by the members of the Bradford Dementia Group (46) and is based on the principles of person centred care developed by prof. Tom Kitwood. (8, 9) The method and coding system have high ecological validity since they were originally developed through ethological observations of many hours in nursing homes, hospital facilities and day care in the UK. (9) The DCM-method is an observational tool that has been used in formal dementia care settings since 1992, both as an instrument for developing person-centred care practice, and as a tool in evaluative research. (11, 12, 38) DCM-method comes with a strict set of guidelines for its implementation.
The results obtained with the DCM-set of validated instruments, used to systematize and quantify observations of resident-staff interactions, can be used in two ways: 1. as a basis for the feed-back to the staff, representing a starting point for the improvement action plans, and 2. repeated measurements serve as a tool to evaluate the effects of the action plans.

I. DCM-8 Measures
1. The Behavioural Category Codes (BCC) describe 24 different domains of participant behaviour that has occurred, with operationalised rules for assignment. (5, 31, 36) (see Table 1). Test-retest reliability is satisfactory (See Systematic review section, below). In addition, a recent pilot study established DCM inter-rater reliability between the researchers, the DCM-trained staff at each site, and the DCM trainer at the first site,
(Concordance coefficient >0.8). (10)

2. Well/Ill Being (WIB) Values - expressed on a six point scale ranging from extreme ill-being (-5) to extreme well-being (+5) (in DCM-8, the criteria for determining -5, -3, -1,+1,+3 and +5 have been better clarified compared to DCM-7). Well and ill being is a global state that cannot be determined by just one 5-minute time-frame. What is now coded in the 5-min frame is an observation of the participants' mood state alongside their level of engagement with their environment. In DCM 8, the 5-min observation
(formerly WIB value) has been renamed ME Value (mood/engagement value). These are shown in Table 2. ME values can be averaged to arrive at a WIB score. This provides an index of relative well-being for a particular time period for an individual or a group. (See Systematic review section for psychometric characteristics.)
Convergent validity is suggested by significant Pearson's Correlation Coefficients in post tests between Individual WIB scores and functional status ($r2=0.571$, $p< 0 .008$), memory ($r2=0.446$, $p<0.007$), and quality of life ($r2=0.617$, $p<0.0001$). This means that low Individual WIB scores are associated with lower physical functional status of residents, lower memory status, and lower quality of life as perceived by close family/carers. (10)

3. Staff behaviours known as Personal Detractions (PDs; 17 types) and Personal Enhencers (PEs) are recorded whenever they occur. Personal Detractions are staff behaviours that have the potential to undermine the personhood of those with dementia (8, 47). These are described and coded according to type and severity. Personal Enhancers have a parallel structure to Personal Detractions (Table 3); they both build on the description of positive person work. The PEs and PDs are further divided around the degree to which they support or undermine the five psychological needs described by Kitwood: comfort, identity, attachment, occupation, inclusion. (8, 9)

II. OTHER MEASURES

Residents:
- Agitation: CMAI
Cohen-Mansfield Agitation Inventory (CMAI)-Long Form (Cohen-Mansfield, 1986; Cohen-Mansfield, Marx, & Rosental, 1989): Seven-point rating scale (1-never observed to 7-observed a few times in an hour) assessing the frequency with which patients manifest up to 29 behaviours associated with agitation, as observed by care staff over the former two weeks. Inter-rater reliability coefficients are high (0.88 to 0.93) and test/re-test reliability is good ($r2=0.74$ to 0.92).(10)
Construct validity of the CMAI Dutch version was established in two independent samples.(48, 49) Factor based symptoms clusters are Physically aggressive, Physically non-aggressive and Verbally agitated behaviour(48). Inter-rater agreement for CMAI items, total score, and cluster scores are 0.22-0.89 (Kappa), 0.89 (Intra Class correlation) and 0.47-0.85 (Inter-rater correlations), respectively. A recent study by the co-applicant S. Zuidema shows high inter-rater coefficients of CMAI total and

clusters scores (Spearman's rho 0.82-0.89) and moderate test-rest reliability of CMAI total and cluster scores (Spearman's rho 0.50-0.73). (Submitted)

- Other neuropsychiatric symptoms: NPI-NH
The NPI–NH is the only nursing home instrument for assessing neuropsychiatric symptoms that has been translated into Dutch (Kat et al., 2002). The NPI–NH
includes 12 neuropsychiatric symptoms: delusions, hallucinations, agitation, depression, anxiety, euphoria/elation, apathy/indifference, disinhibition, irritability, aberrant motor behavior, nighttime disturbances and appetite/eating change. (50) Both the frequency (F) and severity (S) of each symptom are rated on a four- (1–4) and three-point (1–3) Likert scale, respectively. NPI-NH items test-retest correlations range 0.55-0.88, interrater agreement (Kappa statistic) ranges 0.85-1.00, and between raters correlation range 0.173 to 0.914.

- Quality of life - DQoL
Dementia Quality of Life Scale (DQoL) (51) is a 29-item scale which assesses several
domains related to quality of life that were identified as important during focus groups held with people with mild and moderate dementia, carers of people with dementia, and health care workers in dementia. Scales (A, B, C): (A) aesthetics (sum scores 5–25); (B) frequency of feelings: B1: positive affect (sum scores 5–30); B2: negative affect (sum scores 5–55); B3: self esteem (sum scores 5–20); B4: feelings of belonging (sum scores 5–15); and (C) overall quality of life (1–5) (higher scores on quality-of-life subscales indicate better quality of life, except for subscale B2). (52)The scale is found to have good internal consistency, reliability and construct validity.(53)

- RCS - Resident Classification Scale (RCS) (54): Standard assessment conducted by RCS-trained staff for all Australian aged/dementia care residents, to determine their care needs and the relative resource allocation required. Overall care/service need is rated from A (very low) to E (very high) and ratings are used to define categories (1–8 categories). Categories 1 and 2 indicate high care needs, and a higher level of funding. The RCS has good concurrent validity with the Modified Barthel Index with high inter- and intra-rater reliabilities (Spearman's r2=0.84).

Staff
- Stress reactions. The short version of the General Health Questionnaire (GHQ 12) will be used to measure the staff perceived stress ranging from 0-12 (Koeter and Ormel, 1987; Ormel et al., 1989a; 1989b). Ratings pertain to the weeks preceding the administration of the scale. Answer categories are "absent" (0 points), "the same as usual" (0 points), "more that usual "(1 point), and "a lot more that usual" (1 point).

- Job Satisfaction Maastricht Work Satisfaction Scale for Healthcare (MAS-GZ) by Landeweerd et. Al., 1996) consists of 7 subscales with three items each which are rated on a 5-point scale: satisfaction with quality of care, opportunities for self-actualisation/growth, contact with colleagues, contact with residents, satisfaction with supervisor, possibilities for promotion, and clarity of tasks and rules.

III. DESCRIPTION DCM–cycle: OBSERVATIONS - FEED-BACK – ACTION PLAN

OBSERVATION
In brief, an observer (mapper) observes on average five (4-6) residents with dementia continuously over a representative time period (minimal 4 hours a day) in communal areas (living room) of care facilities. After each 5-min period (a time-frame) the above outlined codes (BCC, WIB) are used to record what has happened to each participant and what the behaviour of the staff was in terms of PDs and PEs. (11, 38)

FEED-BACK
In DCM-8, in addition to Personal Detractions (PDs), also the Personal Enhancers (PEs) are been coded, discussed with the staff and related to the residents' ME-values and WIB-scores. Focus group data (11) show that staff appreciated this addition (compared to DCM-7) because they were able to form a complete picture of the participants (residents). Therefore, they were also able to interpret the PD feedback in the context of the lives of participants rather than relating it to themselves in a negative way and possibly becoming defensive. Interestingly, a number of staff teams wanted to keep the strong terminology used in the Personal Detraction coding. Many said that it was this list that had made an impression on them when they first learned about DCM and that it was important not to water this down. This indicates that previously, staff members were possibly not aware of the impact that their behaviour may have on the residents. They seem to appreciate being made aware of this.

ACTION PLANS
Based on feedback discussions, staff draws action plans for improving care on an individual and group level. Action plans are 'vehicles' for the implementation of the principles of person centred care (PCC) in daily practice. The standard principles of PCC are:
- Valuing people with dementia and those who care for them (V);
- Treating people as individuals (I);
- Looking at the world from the perspective of the person with dementia (P);
- A positive social environment in which the person living with dementia can experience relative wellbeing (S); (Brooker, 2004, What is person-centred care in dementia? Reviews in Clinical Gerontology 2004 13; 215–222)

EXAMPLES OF BASIC OBSERVATION – BASED ACTION PLANS:

DCM-Project: Examples of improvement goals;

1. The WIB score of Mss Baker should be improved. Based on her WIB profile, staff develop tailor-made actions to raise her level of engagement in social activities.

2. Positive interactions can be further improved. For instance, when glancing over a magazine together with a resident, other residents can be invited to join in. Hereby, the caregiver may find it useful to choose a place to sit which offers the possibility to survey the level of engagement among the residents.

3. More diversity should be introduced in the choice of activities for the residents.

4. WIB profile suggests that Mss. de Bont may benefit from sensory stimulation interventions (touch, music, taste)

5. The occurrence of Personal Detraction is too high. Action is required in raising the person centred competences of temp personnel.

6. Discuss in a multidisciplinary team the similarities and differences in care needs of the day care group and the residents. Depending on the actual care needs assessment allow for the day care persons to participate in the residents activities and vice versa.

7. Try and see if Mss de Roos will benefit from helping with the household tasks on the PGU.

8. Arrange that a DCM-observer from another unit can observe interactions of the activity coordinator with the Residents.

In sum, Dementia Care Mapping (DCM) is a method in which care improvement plans ('action plans') are based on systematic observations of the actual care as it takes place in formal settings such as nursing homes and day care. The results are fed-back to the staff, which raises their awareness regarding the dependency between their own behaviour and that of the residents. The feed-back occurs in a non-threatening way and does NOT serve as staff evaluation tool. The fact that not only ‘negative’ but also positive events are recorded and brought to light motivates staff to improve their competences and performance. DCM offers a set of tools to be used for the purpose of personal, departmental and organizational development. Through DCM, the staff may attain an important signalling role towards the members of the multidisciplinary care teams in nursing homes (psychologists, nursing home physicians, physiotherapists, occupational therapists). This allows for timely initiation of targeted psychological or other interventions. (12)This is very important in ensuring long-term positive effects of DCM. Furthermore, it is important to emphasise that the DCM-method acts as a channel for a timely implementation of various kinds of improvements, from the individual level (resident, caregivers), to group level (residents, caregivers: eg. professional development needs), PGU-level, multidisciplinary team level, management level and organisation level. This way, the improvement actions become sufficiently tailor-made. A large number of studies on effectiveness of psychosocial interventions for people with dementia and their (in)formal caregivers emphasize the need of individually tailored interventions. (55-57)

Dementia Care Mapping method has been extensively used to improve the quality of care in nursing homes. Australian pilot study established decrease in residents Neuropsychiatric Symptoms (CMAI, RMBPC-disruption), as well as improvement in staff interactions with residents with increase in Positive Care, and Positive Social interactions and decrease in Neutral, Negative Protective, and Negative Restrictive interactions. (10) In the first Dutch pilot study positive effects were obtained for patient well being (WIB-scores), anxiety (GIP), agitation (CMAI), positive staff-resident interactions (PEs), and staff satisfaction with contacts with clients (MAS, VAS). (37)

IV. IN THE PRESENT STUDY, DCM-INTERVENTION COMPONENTS ARE:
1. Dementia Care Mapping (DCM) Organisational Briefing Day - 1-day training course aimed to provide organization-wide basic understanding of the DCM method thereby ensuring endorsement of DCM goals and methods, and assisting in its implementation into an organization or setting.

2. Staff training-intervention group:
- Basic user: a 4-day course teaching basic concepts and skills. A basic user can participate in a DCM team under supervision of advanced user;

- Advanced user: a 3-day course on background and theory of DCM. Advanced user is able to perform care mapping, report observations, lead a DCM-team, give feed-back to the staff and instruct/support them in drawing up action plans. Per organization 1 staff member may become an advanced user while the number of basic users will depend on the number and size of the PGU-units per nursing home.

3. Three DCM-cycles. Description single DCM-cycle: 1. Observing caregiver-patient interactions by the DCM-trained staff over a period of time and recording information about their experience of care; 2. feeding back the results to the staff, 3. staff draw up action plans (in collaboration with other professionals) to bring about changes towards more person-centred care; 4 evaluative observation

E. OUTCOME PARAMETERS
I. Residents: 1. Neuropsychiatric symptoms - primary (CMAI, NPI-NH), 2. Quality of life (WIB, Dqol) – secondary

II. Staff: 1. Job-related stress – secondary (GHQ – short version); 2. Sense of competence – secondary (SCQ); 3. Job-stress related absenteeism, and turnover (organisational databases) -secondary; 4. Job satisfaction (MAS en VAS) - secondary;

SAMPLE SIZE calculation (motivate assumptions) and feasibility of recruitment

According to a recent RCT-study by Chenoweth et al., (2009), with 5 clusters in the control and 5 in DCM group, 20% atrition rate in 8 months, and an average of 14 evaluable patients at follow up, lead to a treatment difference of 10.9. As the 95% confidence interval of the mean difference was 0.7 - 21.1, the standard error of the difference was approximately (21.1- 0.7) /4=5.1.

Therefore, in our study, with similar attrition rate, standard deviation, cluster sizes, ICC, analysis method and design, but with 9 clusters per arm, we would have a standard error of the difference of approximately 5.1 * sqrt(5/9)= 3.8.

Assuming that the true difference between the treatments is 10.9, the power of our study then is 80% (two-sided testing at alpha=0.05).

F. DATA-ANALYSIS and presentation/syntheses
The effects on the primary outcomes will be evaluated using linear mixed effect models with treatment, baseline measure, and control variables (used in the sequential balancing minimization procedure; Borm et al.2005) as covariates and Psycho-geriatric unit (PGUU) as a random effect, to correct for dependencies within PGUs. Structural Equation Modeling will be used in the secondary analysis to evaluate the plausibility of a theoretical model of causal chains of effects based on the suggested mediator variables (e.g. WIB, PE/PD). Additionally, quantitative and qualitative process analyses will be carried out in order to determine the level of use of new knowledge and skills in daily practice, and in order to determine relevant facilitators and barriers to implementing new knowledge. To that aim, checklists will be used.

G. ECONOMIC EVALUATION
This cost effectiveness analysis focuses on adding the DCM-intervention to nursing home Psycho-geriatric Units (PGUs) and comparing this to usual care from a societal perspective. The economic evaluation is based on the general principles of a cost-effectiveness analysis from a societal viewpoint.
Based on the above mentioned primary outcomes that vary between objects of study (residents and staff), two different incremental cost effectiveness ratios (ICERs) will be computed: Costs per quality of life (DQOL) increase (residents), and costs per increase in job satisfaction (staff), Other outcome measures such as neuropsychiatric symptoms relief (residents), work-stress and stress related absenteeism and turnover rate will be monetarized and entered in the ICER on the costs side. Finally, as a scenario, a net monetary benefit will be determined.

Cost analysis:
The cost analysis consists of two main parts. First, on patient level, volumes of care (to determine the incremental direct health care costs) based on the production process of the care program (with and without DCM) will be measured prospectively using an activity based costing approach. Focusing on activities performed with costs accumulated at the activity level(s) of the health care production processes, standardized case report forms will be used to assess time invested by nursing staff (including extra training), psychologist, physician, and activity therapist. Also, number of psychiatric consultations, consultations of medical specialists at hospitals, hospital admissions (number of days in hospital) and use of antidepressant as well as all other medication will be recorded. With regard to staff the costs associated with sick leave (stress related absenteeism) and turnover costs will be collected using diaries and the nursing home administration. Furthermore the DCM intervention costs will be determined. It is hypothesized that the use of the above outlined intervention results in less health care consumption (lower care needs, lower use of (psychoactive) medication, less treatments of adverse events, lower staff illness, absence and turnover rates) compared to usual care. Resident Classification Scale (RCS) (DoHA 1997)(37) will be used to determine and compare change in patients care needs and the relative resource allocation in the treatment and control group. Overall, care/ service needs can be rated from A (very low) to E (very high) and ratings are used to define categories (1–8 categories). When the resident is allocated a high proportion of Cs, Ds & Es, they are rated as a Category 1 or 2, indicating high care nHoieeds, and costs.
Second, the cost prices for each volume of consumption will be determined, to use these for multiplying the volumes registered for each participating patient. The guidelines for cost analyses will be used (CVZ, Oostenbrink et al., 2004). For units of care/ resources where no guideline or standard prices are available, real cost prices will be determined. As a basis for the cost price calculations for each participating unit a unique cost price, based on standardized calculation methods will be determined. This results in total costs incorporating total resident related costs and total staff costs per PGU.

The impact of deterministic variables such as cost prices for volume parameters that are incremental cost drivers, will be investigated using sensitivity analyses on the basis of the range of extremes.

ICERs will be computed and sampling uncertainty will be determined using the bootstrap or Fieller method. Finally, a cost-effectiveness acceptability curve will be derived that is able to evaluate efficiency by different thresholds for the ICERs.

H. SYSTEMATIC REVIEW
Here, we are using the existing systematic review made by our co-applicant prof. Dawn Brooker and published in 2005. (38) In addition, we will discuss studies which appeared between 2005 and 2009, separately. The purpose of this review of the research literature is to answer some key questions about the nature of the DCM-tool and its efficacy.

SEARCH TERMS
- Population
People with dementia residing in nursing homes.

- Intervention
Dementia Care Mapping, DCM

- Methodological filters
Descriptive studies and disertations were not included

DATABASES USED AND NUMBER OF MANUSCRIPTS RETRIEVED
The international DCM network led by the University of Bradford maintains a DCM bibliographic database that contains all known publications on DCM (http://www.bradford.ac.uk/acad/health/dcm). This database formed the basis of this review. It includes refereed and nonrefereed journal articles, book chapters, theses, and non-English language texts. It is updated by the Bradford Dementia Group with
Annual bibliographic searches on Medline, Cinahl, and Psychinfo using the terms ‘Dementia Care Mapping’ and ‘DCM’.
Thirty-four articles met the inclusion criteria. They were divided into five main types: (a) cross-sectional surveys, (b) evaluations of interventions, (c) practice development evaluations, (d) multimethod evaluations, and (e) papers investigating the psychometric properties of DCM.

SELECTION PROCEDURE, VALIDITY ASSESSMENT
Articles were included which specifically examined the efficacy of DCM or in which DCM was a main measure in evaluation or research. There were no exclusion criteria based on quality of scientific design. Articles that were purely descriptive were excluded, as were dissertations. The review includes 34 articles published between 1993 and March 2005.

Selected studies: Ballard et al., 2001; Ballard, O’Brien, Reichelt, & Perry, 2002; Ballard et al., 2004; Ballard, Barnett, 1995; Fossey, Lee, and Ballard, 2002; Younger & Martin, 2000,
Martin & Younger, 2000; Martin and Younger, 2001; Kuhn et al., 2002; Kuhn, Edelman, & Fulton, 2005; Kuhn, Fulton, & Edelman, 2004; Edelman, Fulton, and Kuhn, 2004; Edelman, Kuhn, and Fulton, 2004; Brooker & Duce, 2000; Brooker, 2001; Brooker, Foster, Banner, Payne, and Jackson,1998; Powell, et al., 2002; Innes & Surr, 2001; Perrin, 1997; Potkins et al., 2003; Wilkinson, 1993; Williams & Rees, 1997; Pritchard & Dewing, 2001; Chung, 2004;
Bredin, Kitwood, and Wattis,1995; Maguire & Gosling, 2003; Jarrott& Bruno, 2003; Gigliotti, Jarrott, & Yorgason, 2004; Lintern, Woods, & Phair, 2000a; Wylie, Madjar, & Walton, 2002; Thornton, Hatton, and Tatham, 2004; Williams & Rees; Wylie et al., 2002; Heller, 2004.
Douglass & Johnson, 2002

RESULTS (SECONDARY OUTCOME PARAMETERS; DCM- key questions).

1. DOES DCM MEASURE QUALITY OF CARE AND/OR QUALITY OF LIFE?

Concurrent validity (convergent) of WIB scores with proxy quality-of-life measures.
- Fossey and colleagues (2002) demonstrated a significant correlation between WIB scores and the Blau (1977) proxy measure of quality of life.
- Edelman and colleagues (2004) demonstrated a moderately significant correlation between WIB scores and two staff proxy measures of quality of life—the Quality of Life AD–Staff (Logsdon, Gibbons, McCurry, & Teri, 2000) and the Alzheimer’s Disease-Related Quality of Life (ADRQL; Rabins, Kasper, Kleinman, Black, & Patrick, 1999) in adult day care.
- Edelman and colleagues (2004) study did not demonstrate a correlation between any of these measures compared to direct quality-of-life interviews with a less cognitively impaired subgroup.

2. CAN DIFFERENT MAPPERS USE DCM RELIABLY?
- Only Fossey and colleagues (2002) looked at test–retest reliability. The best correlation was between percentage of +3 and +5, followed by overall WIB score. Significance was more moderate for type of BCC but still at an acceptable level.
- Surr and Bonde-Nielsen (2003) outline the various ways in which reliability can be achieved in routine mapping.

3. DOES DCM SHOW REPRESENTATIVE RELIABILITY ACROSS ALL PEOPLE WITH DEMENTIA?
- There is evidence to suggest that level of dependency is correlated with DCM scores, specifically that low WIB scores are associated with high dependency levels. This has been demonstrated statistically on three different continents (Brooker et al., 1998; Chung, 2004; Edelman et al., 2004; Kuhn et al., 2004; Thornton et al., 2004) using three different measures of dependency.

- Brooker and colleagues (1998) found that the correlation between dependency and WIB score disappeared after three successive cycles of DCM. The authors believed that, by this stage, ways of supporting well-being of participants who were highly dependent
had been better established.

- The evidence that dependency level skews DCM results is strong enough to suggest that a measure of dependency should be routinely taken alongside DCM evaluations so that the results can be scrutinized for this relationship.

4. IS DCM A SUITABLE TOOL FOR RESEARCH?
- Acceptable inter-rater reliability is achievable, and concurrent validity with other proxy measures of quality of life has been demonstrated. Fossey and colleagues (2002) demonstrated internal consistency and test–retest reliability.
- What is clear is that BCCs do not measure real-time estimates of different types of behavior (Thornton et al., 2004).

5. WHAT DO THE SCORES MEAN IN TERMS OF BENCHMARKING?
- Work is currently underway to develop an international database of DCM results to which all international strategic DCM partners would have access. The database should include participant and facilities factors that could be used in stratified analyses, correlational studies, and as adjustment factors.

6. WHAT IS A SIGNIFICANT CHANGE IN SCORES?
- Intervention studies (Brooker & Duce, 2000; Brooker, 2001; Gigliotti et al, 2004; Jarrott & Bruno, 2003) report differences in the range of 0.4 to 1.1, which were all statistically significant. Changes in individual WIB scores, WIB value profiles, and BCC profiles are more variable. Further research is needed to clarify what constitutes a clinically significant change.

7. HOW LONG SHOULD A MAP BE?
- Fossey and colleagues (2002) found a statistically significant correlation between the hour prior to lunch and a 6-hr map on all their key indicators at the group level. It is likely that there would be a great deal more variation on an individual level.
- An unpublished U.S study (Douglass & Johnson, 2002) mapped 18 residents during a 6-day period for periods of 2, 4, 6, and 8 hr in a continuing care retirement community. Acceptable levels of interrater reliability were demonstrated in maps of more than 4 hr in duration. This important issue requires further research.

SUMMARY AND CONCLUSION
“These studies report evidence that DCM has a role in practice development and research within the broad aim of improving the quality of life for people with dementia. A large international database on DCM results would help clarify the relationship between DCM results, dependency, diagnostic group, and facility characteristics. Steps need to be taken through the development of the method, training, and accreditation to ensure reliability. The published work on DCM is of variable quality but is growing in strength. DCM’s advantages are that it is standardized, quality controlled, international, responsive to change, multidisciplinary, and has an increasing research base. DCM provides a shared language and focus across professional disciplines, care staff, and management teams. It is seen as a valid measure by frontline staff as well as those responsible for managing and commissioning care. It also provides a shared language between practitioners and researchers. DCM holds a unique position in relation to quality
of life in dementia care, being both an evaluative instrument and a vehicle for practice development in person-centered care. Maintaining a dialogue between the worlds of research and practice in health and social care is a major challenge. DCM provides an opportunity to do this. “(38)

NEW SEARCH; STUDIES 2005-2009
The same search was performed in PubMed on 26-01-2009. 8 studies were retrieved whereof 5 were selected as relevant. Of the remaining three studies, one was in Japanese, one was a neurological study and one concerned hospital care. To the 5 selected studies we have added a Dutch pilot study (PC) which is not retrievable from PubMed. The main findings of the in total 6 studies will be briefly summarized below.

1. “Inappropriate treatment of people with dementia in residential and day care.” Objective - to investigate inappropriate treatment and its context in the care of people with dementia. Method - 85 clients across eight care units providing dementia care were observed by means of a structured Dementia Care Mapping method (DCM). Results - Of the 17 DCM categories for inappropriate treatment, withholding, invalidation, and objectification were the categories coded most frequently. Inappropriate treatment episodes were mostly associated with eating and situations in which a client had a need or request. Most episodes occurred accidentally. Conclusion - The results show that nurses involved in dementia care need more knowledge of how illnesses causing dementia change the way the affected person experiences reality, and of the ethical aspects of their work. (58)
2. “Determining the efficacy of Dementia Care Mapping as an outcome measure and a process for change: A pilot study” Report of a pilot study conducted with 35 dementia care residents in three secure residential care units in the state of New South Wales, Australia, prior to the controlled trial. Aims - To determine the sensitivity of DCM against the validated baseline and outcome measures selected for the trial and to evaluate the utility of the research plan. Results - The utility of the research plan was established. Whilst a significant improvement was shown in staff interactions with residents over time, there was no evidence that DCM improved the residents’ quality of life and well-being in relation to physical and cognitive functioning. There was a reduction in the residents’ levels of agitation (CMAI) and depression. While as a research tool DCM needs further refinement to match the strength of validated outcome measures that more accurately assess the residents’ well-being, DCM procedures improved staff’s attention to monitoring and attending to the residents’ well-being. (10)

3. “Initial validation of DCM 8 in UK field trials” Objectives - To describe DCM 8 and report on the initial validation study of DCM 8.
Results - At a group score level, WIB scores and spread of Behavioural Category Codeswere very similar, suggesting that group scores are comparable between DCM7 and 8. Interviews with mappers and focus groups with staff teams suggested that DCM8 was preferable to DCM 7th edition because of the clarification and simplification of codes; the addition of new codes relevant to person-centred care; and the replacement of Positive Events with a more structured recording of Personal Enhancers. Conclusions - DCM 8 appears comparable with DCM 7th edition in terms of data produced and is well received by mappers and dementia care staff. (11)

4. “Dementia care mapping as a research tool.”
Review. Descriptive data from several different research teams on the distribution and psychometric properties of DCM data were compiled and summarized. Conclusions - Despite the identified limitations, DCM has promise as a research measure, as it may come closer to rating QOL from the perspective of persons with dementia than other available measures. Its utility will depend on the manner in which it is applied and an appreciation of the measure’s strength and limitations. Possible changes that might improve the reliability, validity, and practicality of DCM as a research tool include coding the predominant event

(rather than the 'best' event), shortening the observation period, and adding '0' as a neutral WIB coding option. (12)

5. "Evaluating the quality of life of long-term care residents with dementia."
Review. Resident cognitive impairment, functional impairment, social withdrawal, agitation, depressive symptoms, and number of comorbid conditions had significant negative associations with WIB scores, whereas resident age, gender, and race did not. Data from the UK sample demonstrated a significant relationship between WIB scores and the Care Dependency Scale (Dijkstra et al., 2000) ($r2=0.35$; $p<0.011$), with 14% of the variance in WIB scores accounted for by differences in dependency. (59)

5. "Effects of Dementia Care Mapping (DCM) on the Quality of Life of the nursing home Residents with dementia and on the jos satisfaction of their formal caregivers at Psycho-geriatric Units."
Dutch pilot study investigated pre-post differences (no control group) in behaviour (GIP), agitation (CMAI), and wellbeing (PGCARS). Significant positive effects were found on Orientation (GIP, $p< .05$), and verbale agitation (CMAI; $p<.05$). In addition, significant changes were found in General job satisfaction (VAS; $p< .05$).

I. TIME SCHEDULE: GEDETAILLERERDE FASERING

Months 1-12
1. Symposium with workshops for nursing home staff and managers, and professional organizations in order to acquaint them with the method and raise motivation for their participation in the study;
2. Recruitment and selection of nursing homes, randomisation of eligible PGUs;
3. DCM training (see Intervention components 1 and 2) for organisations and staff

Months 13 - 24
1. Baseline measurement
2. 4 months DCM-cycle: Observation + Feed-back + Action plan
3. T-1 Measurement
4. 4 months DCM-cycle: Observation + Feed back + Action plan
5. T-2 Measurement

Months 25-36
1. Analyses and reporting of the results;
2. Enrolling wait list control group.
3. Closing symposium for nursing homes and professional organizations to present the results and develop a broad implementation strategy.

Expertise, voorgaande activiteiten en producten / Expertise, prior activities and products

The expertise of several research departments is used in this project: Alzheimer Centres Nijmegen, Amsterdam and Maastricht united under ARN, IQ healthcare, several UMC St Radboud departments (Nursing Home sciences, Geriatrics, Nursing Sciences, Medical Technical Assessment), Bradford Dementia Group, University of Bradford, School of Health Studies, UK. In addition, we collaborate closely with the nursing home Kalorama, Beek-Ubbergen, and Friese Wouden and their DCM-team (first DCM-site in NL).
Presently, the principal investigator has 2 PhD students and is supervising 2 PhD students on complex ACN-projects that are related to the current proposal:

1. LDP-Gelderland which includes divers research and management activities such as developing methods and instruments to support regional dementia networks in evaluating efficacy of different improvement projects, complex analyses of the findings, close collaboration with regional LDP-coordinators and work groups, dissemination of knowledge and expertise accumulated at UMCN St Radboud to support regional efforts in improving dementia care, proactive approach to meet the needs of the regions timely, taking care that as much as possible knowledge is accumulated about this kind of
large projects in order to be able to use it in future collaborative efforts in health care.

2. EASYcare /GIDS: Includes development of an EASYcare-based Dementia Training Program (DTP) aimed at stimulating collaboration in dementia primary care. The programme is aimed at increasing the number of cognitive assessments and dementia diagnoses, and at improving attitudes and knowledge of GPs and nurses. The DTP is a complex educational intervention and consists of workshops, coaching program, case-based consultations, internet forum, and a Computerized Clinical Decision Support
System on dementia diagnostics and management. Experiences from these two projects are highly compatible with the requirements of the presently proposed project. Earlier projects include: development of quality indicators for the memory clinics, modeling and evaluation of small group learning at UMC St Radboud Nijmegen (38), research on
semantic memory and semantic interpretation (dissertation), all relevant for the present project.

Dr. I. Draskovic, prof. D. Brooker and Ms. A. Post are all members of the Advising Council ('Raad van Advies') for broad implementation of DCM in the Netherlands.

Prof. Myrra Vernooij-Dassen (PhD) is principal investigator in the Nijmegen Centre for Evidence Based Practice and director of Nijmegen Alzheimer Centre. She has a chair on psychosocial aspects of care for frail elderly people. She is affiliated to the

scientific Institute of Quality of healthcare and the department of nursing home medicine of the Radboud University Nijmegen Medical centre and to the Kalorama foundation She is trained in sociology and research methodology and has performed a large body of research on quality of care. She is author of more than 100 scientific and professional publications and supervises twenty PhD students. She is co-chair of Interdem, a pan-European research group on detection and timely INTERvention in DEMentia. She is honorary visiting professor of the School of Health of Bradford University. Prof. Vernooij-Dassen is a renowned expert in the field of research on psychosocial interventions in dementia care.

Professor Brooker qualified as a clinical psychologist at the University of Birmingham in the UK in 1984. She has demonstrated both academic and clinical achievement and leadership in the field of dementia studies. Specifically, she is recognised for scholarship in practice development of person-centred care for people with dementia and in innovative therapeutic interventions. She is currently undertaking a cluster randomised controlled trial of supporting older people with mental health problems in extra care housing utilising the Enriched Opportunities Programme. Professor Brooker's work with CSCI (Commission for Social Care Inspection) has culminated in a new observational method and training for Inspection staff reviewing care homes. The tool (SOFI – Short Observation Framework for Inspection) is currently being incorporated into all inspections of dementia specific care homes in England. A thematic large-scale survey of care-homes for people with dementia is planned using SOFI. Professor Brooker's recent book on Person Centred Dementia Care published in 2007 has redefined and revitalised the meaning of person centred for people with dementia, their families and service providers.

Project members all have an excellent and long standing record in research on quality of care in general, quality of care in dementia, specialization in nursing home medicine, geriatrics, implementation of DCM in nursing home staff interventions (see: http://www.wokresearch.nl, www.umcn.nl/vphg, http://www.brad.ac.uk/acad/health/dementia/). The two MTA researchers have both a profound scientifical background in a basic discipline. E. Adang (Health Economist) has 13 international publications and some national publications in the field of economics. He is also scientific reviewer for the Dutch programme 'Doelmatigheids Onderzoek'. Rogier Donders (medical statistics) is an expert in the field of medical statistics and a scientific reviewer with several international publications.

Publicaties / Publications

- Draskovic; I, Vernooij-Dassen M, Verhey F, Scheltens P, Olde Rikkert M.
Development of quality indicators for memory clinics. Int J Geriatr Psychiatry. 2008 Feb;23(2):119-28.

- Draskovic; et al. Kwaliteitsindicatoren geheugenpoliklinieken. Modern Medicine, 2008
- Olde Rikkert M, Vernooij-Dassen M, Draskovic I, Verhey FRJ, Jolles J, Gillissen F, Scheltens P. Verpleeghuis te snel in beeld. Medisch Contact 2005;22:931-933.

- Perry M, Melis RJ, Teerenstra S, Draskovic; I, van Achterberg T, van Eijken MI, Lucassen P, Olde Rikkert M. An in-home geriatric programme for vulnerable community-dwelling older people improves the detection of dementia in primary care. Int J Geriatr Psychiatry. 2008 Dec;23(12):1312-9.

- Scholzel-Dorenbos C, Draskovic I, Vernooij-Dassen M, Olde Rikkert M.
Quality of Life and Burden of Spouses of Alzheimer Disease Patients. Alzheimer Disease and Associated Disorders, (electr publ. ahead of print) Nov. 2008

- Perry M, Draskovic; I, van Achterberg T, Borm GF, van Eijken MI, Lucassen P, Vernooij-Dassen MJ, Olde Rikkert MG. Can an EASYcare based dementia training programme improve diagnostic assessment and management of dementia by general practitioners and primary care nurses? The design of a randomised controlled trial. BMC Health Serv Res. 2008 Apr 2;8:71.

- Olde Rikkert M, Draskovic I, Vernooij-Dassen, M. The planning of appropriate medical and social care in dementia. In: Waldemar G, Burns A (Eds): Alzheimer disease. (In press) Oxford: University Press

- Graff MJ, Adang EM, Vernooij-Dassen MJ, Dekker J, Jönsson L, Thijssen M, Hoefnagels WH, Rikkert MG. Community occupational therapy for older patients with dementia and their care givers: cost effectiveness study. BMJ. 2008 Jan 19;336(7636):134-8. Epub 2008 Jan 2.

- Graff MJ, Vernooij-Dassen MJ, Thijssen M, Dekker J, Hoefnagels WH, Rikkert MG.
Community based occupational therapy for patients with dementia and their care givers: randomized controlled trial. BMJ. 2006 Dec 9;333(7580):1196. Epub 2006 Nov 17.

- Moniz-Cook E, Vernooij-Dassen M, Woods R, Verhey F, Chattat R, De Vugt M, Mountain G, O'Connell M, Harrison J, Vasse E, Dröes RM, Orrell M; INTERDEM group.
A European consensus on outcome measures for psychosocial intervention research in dementia care. Aging Ment Health. 2008 Jan;12(1):14-29.

- Spijker A, Vernooij-Dassen M, Vasse E, Adang E, Wollersheim H, Grol R, Verhey F. Effectivenss of nonpharmacological interventions in delaying institutionalization of patients with dementia: a meta-analysis. J Am Geriatr Soc. 2008 Jun;56(6):1116-28.

- Graff MJL, Vernooij-Dassen, MJM, Thijssen M, Dekker J, Hoefnagels WHL, Olde Rikkert MGM. Effects of community occupational therapy on quality of life, mood and health status in dementia patients and their caregivers: a randomized

controlled trial, Journals of Gerontology: Medical Science. J Gerontol A Biol Sci Med Sci. 2007 ;62(9):1002-9.

Gerritsen, D. L., Achterberg, W. P. Steverink, N. Pot, A. M. Frijters, D. H. Ribbe, M. W. The MDS Challenging Behavior Profile for long-term care. Aging Ment Health, 2008; 12(1): 116-23

van Achterberg T, Peijnenburg R, van Eijken MI. Omgaan met gedragsproblemen bij patiënten met dementie (niet-medicamenteuze aanbevelingen) Richtlijn voor verpleegkundigen en verzorgenden. Nijmegen; 2008.

Zuidema S, Koopmans R, Verhey F. Prevalence and predictors of neuropsychiatric symptoms in cognitively impaired nursing home patients. J Geriatr Psychiatry Neurol 2007;20(1):41-9.

Referenties / References

1. Ballard C, et al. Quality of life for people with dementia living in residential and nursing home care. Int Psychogeriatr 2001;13(1):93-106.
2. Fossey J, Ballard C, et al. Effect of enhanced psychosocial care on antipsychotic use in nursing home residents with severe dementia; RCT. BMJ 2006;332(7544):756-61.
3. Zuidema S, Koopmans R, Verhey F. Prevalence and predictors of neuropsychiatric symptoms in cognitively impaired nursing home patients. J Geriatr Psychiatry Neurol 2007;20(1):41-9.
4. Verpleeghuizen garanderen minimale zorg niet. Den Haag: Inspectie voor de gezondheidszorg; 2004.
5. Inspectorate DHC. Inspectie waarschuwt verzorgingshuizen. In: De Volkskrant; 2008.
6. Bostick J, et al. Systematic review of studies of staffing and quality in nursing homes. J.Am.Med.Dir.Assoc. 2006;7:336-376.
7. Zichtbare Zorg: Verpleging v, thuiszorg. De toon gezet: één taal voor kwaliteit. Den Haag: Stuurgroep Kwaliteitskader Verantwoorde Zorg; 2008.
8. Kitwood T. Toward a theory of dementia care: ethics and interaction. J Clin Ethics 1998;9(1):23-34.
9. Kitwood T, Bredin K. Towards a theory of dementia care: personhood and well-being. Ageing Soc 1992;12:269-87.
10. Chenoweth L, Jeon YH. Determining the efficacy of Dementia Care Mapping as an outcome measure and a process for change: A pilot study. Aging Ment Health 2007;11(3):237-45.
11. Brooker D, Surr C. Dementia Care Mapping (DCM): Initial validation of DCM 8 in UK field trials. International Journal of Geriatric Psychiatry 2006;21:1-8.
12. Sloane PD, Brooker D, et al. Dementia care mapping as a research tool. Int J Geriatr Psychiatry 2007;22(6):580-9.
13. Edvardsson D, Winblad B, Sandman PO. Person-centred care of people with severe Alzheimer's disease. Lancet Neurol 2008;7(4):362-7.
14. van Weert JC, et al. The effects of the implementation of snoezelen on the quality of working life in psychogeriatric care. Int Psychogeriatr 2005;17(3):407-27.
15. van Weert JC, et al. Behavioral and mood effects of snoezelen integrated into 24-hour dementia care. J Am Geriatr Soc 2005;53(1):24-33.
16. van Weert JC, et al. Effects of snoezelen, integrated in 24 h dementia care, on nurse-patient communication during morning care. Patient Educ Couns 2005;58(3):312-26.
17. NVVA. Handreiking ‘Multidisciplinair werken aan Probleemgedrag’. 2009.
18. van Achterberg T, et al. Omgaan met gedragsproblemen bij patiënten met dementie. Richtlijn. Nijmegen; 2008.
19. CBS. Gezondheid en zorg in cijfers 2007. Den Haag: CBS; 2007 2007.
20. de Lange J, et al. Hoe vaak komt dementie voor en hoeveel mensen sterven eraan? Bilthoven: RIVM; 2003.
21. Ballard CG, et al. A 1-year follow-up study of behavioral and psychological symptoms in dementia among people in care environments. J Clin Psychiatry 2001;62(8):631-6.
22. Brodaty H, et al. Nursing home staff attitudes towards residents with dementia: strain and satisfaction with work. J Adv Nurs 2003;44(6):583-90.
23. van der Kwartel A, et al. Arbeid in Zorg en Welzijn. Utrecht: Prismant; 2008.
24. Maslach Ch SW, Leiter MP. Job burnout. Annu. Rev. Psychol. 2001;52:397-422.
25. Meijer A, Van Campen C, Kerkstra A. A comparative study of the financing, provision and quality of care in nursing homes. J Adv Nurs 2000;32(3):554-61.
26. Vernooij-Dassen et al. Zorg voor mensen met dementie en arbeidsmarkt. Arbeidsmarkt en zorgvraag. Den Haag: RVZ; 2006.
27. Paquay L, et al. Tasks performance by registered nurses in nursing homes. Int J Nurs Stud 2007;44(8):1459-67.
28. Actiz. Arbeidsmarkt verpleging, verzorging en thuiszorg.
29. Dementia. The Hague: Health Council of the Netherlands; 2002.
30.The labour market and the demand for care. Dutch Council for Public Health and Health Care; 2006.
31. Hoeffer B, et al. Assisting cognitively impaired nursing home residents with bathing. Gerontologist 2006;46(4):524-32.
32. Rader J, et al. The bathing of older adults with dementia. Am J Nurs 2006;106(4):40-8, quiz 48-9.
33. Koningsveld E, et al. National costs of working conditions for workers in the Netherlands 2001 The Hague: Ministry of Social Affairs and Employment; 2003.
34. Brooker D, et al. Enriching Opportunities for People living with Dementia in Nursing Homes. Age Ment Health 2007;11(4):361-370.
35. Woolley RJ, et al. The feasibility of care mapping to improve care for physically ill older people in hospital. Age Ageing 2008;37(4):390-5.
36. Verkaik R, et al. The effects of psychosocial methods on depressed, aggressive and apathetic behaviors of people with dementia Int J Geriatr Psych 2005;20(4):301-14.
37. Kuiper D DG. Dych DCM pilot study Rijksuniversiteit Groningen (RuG); 2007.

38. Brooker D. Dementia Care Mapping (DCM): A review of the research literature. The Gerontologist 2005;45 (Special no. 1(1)):11-18.
39. Parent-Thirion A ME, Hurley J, Vermeylen G. Fourth European Working Conditions Survey. Dublin: European Foundation for the Improvement of Living and Working Conditions; 2007.
40. EWCS. Work-Related Stress. 2005. Report No.: EF/05/127/EN.
41. VENVN. Peiling werkdruk: werken in de zorg pittig! 2009 [cited 2009 25 Jan]; Available from: http://www.venvn.nl/
42. Eerste resultaten Nationale Enquête Arbeidsomstandigheden: TNO Arbeid; 2004.
43. Zuidema SU, et al. Prevalence of neuropsychiatric symptoms in .. Dutch nursing home patients with dementia. Int J Geriatr Psychiatry 2007;22(7):632-8.
44. Suzuki M, et al. DCM-J Nippon Ronen Igakkai Zasshi 2008;45(1):68-76.
45. Borm G, et al. Sequential balancing: allocation in clinical trials. Contemp Clin Trials 2005;26(6):637-45.
46. Bradford_Dementia_Group. The DCM Method (7th ed.). University of Bradford; 1997.
47. Kitwood T. Professional and moral development for care work. J Moral Educ 1998;27(3):401-11.
48. Zuidema SU, et al. Agitation in Dutch NH residents with dementia: CMAI. Dement Geriatr Cogn Disord 2007;23(1):35-41.
49. de Jonghe JF, Kat MG. Factor structure and validity of the CMAI-Dutch. J Am Geriatr Soc 1996;44(7):888-9.
50. Zuidema SU, et al. Neuropsychiatric Symptoms in Nursing Home Patients. Dement Geriatr Cogn Disord 2007;24(3):169-176.
51. Brod M, Stewart AL, Sands L, Walton P. Conceptualization and measurement of quality of life in dementia:DQoL. Gerontologist 1999;39(1):25-35.
52. Graff MJ, Vernooij-Dassen MJ, Thijssen M, Dekker J, Hoefnagels WH, Olderikkert MG. Effects of community occupational therapy on quality of life, mood, and health status in dementia patients and their caregiversJ Gerontol A Biol Sci Med Sci 2007;62(9):1002-9.
53. Selwood A, Thorgrimsen L, Orrell M. Quality of life in dementia--a one-year follow-up study. Int J Geriatr Psychiatry 2005;20(3):232-7.
54. The Resident Classification Scale The Residential Care Manual. Canberra: Department of Health and Aged Care; 1997.
55. Ulstein ID, et al. A one-year randomized controlled psychosocial intervention study.. Dement Geriatr Cogn Disord 2007;24(6):469-75.
56. von Gunten A, et al. Vocally disruptive behavior in the elderly. Int Psychogeriatr 2008;20(4):653-72.
57. Opie J, Doyle C, O'Connor DW. Challenging behaviours in nursing home residents with dementia. Int J Geriatr Psychiatry 2002;17(1):6-13.
58. Sormunen S, et al. Inappropriate treatment of people with dementia in residential and day care. Aging Ment Health 2007;11(3):246-55.
59. Sloane PD, et al. Evaluating the quality of life of long-term care residents with dementia. Gerontologist 2005;45 Spec No 1(1):37-49.

Financiële gegevens / Financial data

ZonMw budget

	Jaar / Year								
**Kostenpost**	*1*	*2*	*3*	*4*	*5*	*6*	*7*	*8*	**Totaal / Total**
Personeel	87,311	86,803	96,189	0	0	0	0	0	270,303
Materieel	16,500	12,500	16,700	0	0	0	0	0	45,700
Implementatie	0	0	0	0	0	0	0	0	0
Apparatuur	0	0	0	0	0	0	0	0	0
Overig	0	0	0	0	0	0	0	0	0
**Totaal / Total**	**103,811**	**99,303**	**112,889**	**0**	**0**	**0**	**0**	**0**	**316,003**

Co-financiering / Cofinancing

Naam co-financier / Name of cofinancier	Bedrag / Amount	Status

Bijzondere gegevens / Additional information

Vergunningen / Permits

	Vergunning nodig / Permit required?		Vergunning verkregen / Permit obtained?	
	Ja / Yes	Nee / No	Ja / Yes	Nee / No
METC		X		X
DEC		X		X
WBO		X		X

Onderschrijvingen / Assents

	Ja / Yes	Nee / No
Code biosecurity / Code Biosecurity		X
Code openheid dierproeven / Code Transparency of Animal Testing		X

Andere vergunningen / Other permits

Th METC permit will be requested as soon as possible.
De METC vergunning wordt zo snel mogelijk aangevraagd.

Ondertekening / Signatures

Naam projectleider en penvoerder:<br>I Draskovic	Naam bestuurlijk verantwoordelijke:<br>EARJ Lohman
Plaats en datum:	Plaats en datum:
Handtekening:	Handtekening:

Ronde 2010 -2012 DoelmatigheidsOnderzoek

BEGROTINGSOVERZICHT

Let op: dit overzicht niet invullen, enkel de gearceerde velden in de achterliggende tabbladen
Naam aanvrager en projecttitel invullen op het tabblad 'toelichting'
Deze overzichtspagina vult zich dan vanzelf!!!

Aanvraagnummer: 80-82310-97-10061
Dr. Mw I. Draskovic
An RCT study of (cost) effectiveness of Dementia Care Mapping intervention in nursing home settings

	2010	2011	2012	2013	2014	Totaal
1 Personele kosten	€ 87.311	€ 86.803	€ 96.189	-	-	€ 270.304
2 Materiële kosten	€ 16.500	€ 12.500	€ 16.700	-	-	€ 45.700
3 Implementatiekosten	-	-	-	-	-	-
4 Apparatuurkosten	-	-	-	-	-	-
5 Overige kosten	-	-	-	-	-	-
Totale lasten	€ 103.811	€ 99.303	€ 112.889	€ 0	€ 0	€ 316.004
MINUS: 6 Bijdragen van eigen instelling c.q.derden	-	-	-	-	-	-
**Aangevraagd budget bij ZonMw**	€ 103.811	€ 99.303	€ 112.889	€ 0	€ 0	**€ 316.004**

Ronde 2010 -2012 DoelmatigheidsOnderzoek

BEGROTINGSOVERZICHT

**Aanvrager:**	Dr. Mw I. Draskovic
**Projecttitel:**	An RCT study of (cost) effectiveness of Dementia Care Mapping intervention in nursing home settings
**Projectnummer:**	Aanvraagnummer: 80-82310-97-10061

Toelichting

ZonMw

Aanvraagnummer: 80-82310-97-10061
Dr. Mw I. Draskovic
An RCT study of (cost) effectiveness of Dementia Care Mapping intervention in nursing home settings

Enkel kolommen B, C, D en E invullen. De overige kolommen worden dan vanzelf gevuld.
* Zie de ZonMw subsidievoorwaarden, Bijlage A, Richtlijnen voor het opzetten van de begroting. Het percentage van 16% geldt voor universitaire instellingen.
Op het tabblad 'toelichting' dient u aan te geven hoe u tot een ander percentage dan de 16% bent gekomen.

1.a PERSONELE KOSTEN 2010

nr	Functie	Schaal	Bruto maand salaris	% fte inzet	Aantal mnd	Salariskosten	Sociale lasten 37%	Bruto-salariskosten	Opslag % overhead 16% = universiteit	Bedrag overhead	Totaal
1	Projecbegeleiding Mver	16.03	0	-	0	€ 0	€ 0	€ 0	16%	€ 0	€ 0
2	Projecbegeleiding IDra	11.08	4.135	0,200	12	€ 9.925	€ 3.672	€ 13.597	16%	€ 2.176	€ 15.773
3	Junior onderzoeker	10.05	2.996	0,800	12	€ 28.763	€ 10.642	€ 39.406	16%	€ 6.305	€ 45.710
4	Onderzoeksassistentie	08.05	2.758	0,400	12	€ 13.237	€ 4.898	€ 18.135	16%	€ 2.902	€ 21.036
5	Methodoloog	12.10	5.025	0,050	6	€ 1.507	€ 558	€ 2.065	16%	€ 330	€ 2.396
6	Statisticus	12.10	5.025	0,050	6	€ 1.507	€ 558	€ 2.065	16%	€ 330	€ 2.396
7	0	0	0	-	0	€ 0	€ 0	€ 0	16%	€ 0	€ 0
8						-	-	-		-	-
9											
..											
**Totaal personele kosten 2010**						**€ 54.940**	**€ 20.328**	**€ 75.268**		**€ 12.043**	**€ 87.311**

1.b PERSONELE KOSTEN 2011

nr	Functie	Schaal	Bruto maand salaris	% fte inzet	Aantal mnd	Salariskosten	Sociale lasten 37%	Bruto-salariskosten	Opslag % overhead 16% = universiteit	Bedrag overhead	Totaal
1	Projecbegeleiding Mver	16.04	0	-	0	€ 0	€ 0	€ 0	16%	€ 0	€ 0
2	Projecbegeleiding IDra	11.09	4.335	0,200	12	€ 10.404	€ 3.850	€ 14.254	16%	€ 2.281	€ 16.534
3	Junior onderzoeker	10.06	3.170	0,800	12	€ 30.430	€ 11.259	€ 41.689	16%	€ 6.670	€ 48.359
4	Onderzoeksassistentie	08.06	2.872	0,400	12	€ 13.787	€ 5.101	€ 18.889	16%	€ 3.022	€ 21.911
5				-		-	-	-	16%	-	-
6				-		-	-	-	16%	-	-
7	0	0	0	-	0	€ 0	€ 0	€ 0	16%	€ 0	€ 0
8						-	-	-		-	-
**Totaal personele kosten 20011**						**€ 54.621**	**€ 20.210**	**€ 74.831**		**€ 11.973**	**€ 86.803**

1.c PERSONELE KOSTEN 2012

nr	Functie	Schaal	Bruto maand salaris	% fte inzet	Aantal mnd	Salariskosten	Sociale lasten 37%	Bruto-salariskosten	Opslag % overhead 16% = universiteit	Bedrag overhead	Totaal
1	Projecbegeleiding Mver	16.09	0	-	0	€ 0	€ 0	€ 0	16%	€ 0	€ 0
2	Projecbegeleiding IDra	12.08	4.538	0,200	12	€ 10.890	€ 4.029	€ 14.920	16%	€ 2.387	€ 17.307
3	Junior onderzoeker	10.07	3.347	0,800	12	€ 32.130	€ 11.888	€ 44.018	16%	€ 7.043	€ 51.061
4	Onderzoeksassistentie	08.06	2.994	0,400	12	€ 14.370	€ 5.317	€ 19.686	16%	€ 3.150	€ 22.836
5	Methodoloog	12.10	5.228	0,050	6	1.568,34	580,29	€ 2.149	16%	€ 344	€ 2.492
6	Statisticus	12.10	5.228	0,050	6	1.568,34	580,29	€ 2.149	16%	€ 344	€ 2.492
7	0	0	0	-	0	€ 0	€ 0	€ 0	16%	€ 0	€ 0
8						-	-	-		-	-
**Totaal personele kosten 2012**						**€ 60.527**	**€ 22.395**	**€ 82.922**		**€ 13.267**	**€ 96.189**

1.d PERSONELE KOSTEN 2013

nr	Functie	Schaal	Bruto maand salaris	% fte inzet	Aantal mnd	Salariskosten	Sociale lasten 37%	Bruto-salariskosten	Opslag % overhead 16% = universiteit	Bedrag overhead	Totaal
1						€ 0	€ 0	€ 0	16%	€ 0	€ 0
2						€ 0	€ 0	€ 0	16%	€ 0	€ 0
3						€ 0	€ 0	€ 0	16%	€ 0	€ 0
4						€ 0	€ 0	€ 0	16%	€ 0	€ 0
5						€ 0	€ 0	€ 0	16%	€ 0	€ 0
6						€ 0	€ 0	€ 0	16%	€ 0	€ 0
7						€ 0	€ 0	€ 0	16%	€ 0	€ 0
8						€ 0	€ 0	€ 0	16%	€ 0	€ 0
**Totaal personele kosten 2013**						**€ 0**	**€ 0**	**€ 0**		**€ 0**	**€ 0**

1.e PERSONELE KOSTEN 2014

nr	Functie	Schaal	Bruto maand salaris	% fte inzet	Aantal mnd	Salariskosten	Sociale lasten 37%	Bruto-salariskosten	Opslag % overhead 16% = universiteit	Bedrag overhead	Totaal
1						€ 0	€ 0	€ 0	16%	€ 0	€ 0
2						€ 0	€ 0	€ 0	16%	€ 0	€ 0
3						€ 0	€ 0	€ 0	16%	€ 0	€ 0
4						€ 0	€ 0	€ 0	16%	€ 0	€ 0
5						€ 0	€ 0	€ 0	16%	€ 0	€ 0
6						€ 0	€ 0	€ 0	16%	€ 0	€ 0
7						€ 0	€ 0	€ 0	16%	€ 0	€ 0
8						€ 0	€ 0	€ 0	16%	€ 0	€ 0
**Totaal personele kosten 2014**						**€ 0**	**€ 0**	**€ 0**		**€ 0**	**€ 0**

Ronde 2010 -2012 DoelmatigheidsOnderzoek

Aanvraagnummer: 80-82310-97-10061
Dr. Mw I. Draskovic
An RCT study of (cost) effectiveness of Dementia Care Mapping intervention in nursing home settings

**2.a MATERIELE KOSTEN 2010**	**Totaal**
congres/scholingskosten	€ 2.000
Reiskosten instellingen	3.500,00
trainerskosten	10.000,00
Internationale Project Meetings	1.000,00
**Totaal materiële kosten 2010**	**€ 16.500**

**2.b MATERIELE KOSTEN 2011**	**Totaal**
congres/scholingskosten	€ 2.000
Publicatie/vertaalkosten/proefschrift	€ 1.500
Reiskosten instellingen	€ 2.000
trainerskosten	€ 6.000
Internationale Project Meetings	€ 1.000
	-
	-
**Totaal materiële kosten 2011**	**€ 12.500**

**2.c MATERIELE KOSTEN 2012**	**Totaal**
congres/scholingskosten	€ 2.000
Publicatie/vertaalkosten/proefschrift	€ 1.200
druk/printing/porto/vragenlijstkosten	€ 7.500
trainerskosten	€ 4.000
Internationale Project Meetings	€ 2.000
**Totaal materiële kosten 2012**	**€ 16.700**

**2.d MATERIELE KOSTEN 2013**	**Totaal**
**Totaal materiële kosten 2013**	**€ 0**

**2.e MATERIELE KOSTEN 2014**	**Totaal**
**Totaal materiële kosten 2014**	**€ 0**

ZonMw

Ronde 2010 -2012 DoelmatigheidsOnderzoek

Aanvraagnummer: 80-82310-97-10061
Dr. Mw I. Draskovic
An RCT study of (cost) effectiveness of Dementia Care Mapping intervention in nursing home settings

3.a IMPLEMENTATIEKOSTEN 2010	Totaal
	-
	-
	-
	-
Totaal implementatiekosten 2010	-

3.b IMPLEMENTATIEKOSTEN 2011	Totaal
	-
	-
	-
	-
Totaal implementatiekosten 2011	€ 0

3.c IMPLEMENTATIEKOSTEN 2012	Totaal
	-
	-
	-
	-
Totaal implementatiekosten 2012	€ 0

3.d IMPLEMENTATIEKOSTEN 2013	Totaal
	-
	-
	-
Totaal implementatiekosten 2013	€ 0

,

3.e IMPLEMENTATIEKOSTEN 2014	Totaal
	-
	-
	-
Totaal implementatiekosten 2014	€ 0

ZonMw

Ronde 2010 -2012 DoelmatigheidsOnderzoek

Aanvraagnummer: 80-82310-97-10061
Dr. Mw I. Draskovic
An RCT study of (cost) effectiveness of Dementia Care Mapping intervention in nursing home settings

* zie voor afschrijvingspercentages de ZonMw subsidievoorwaarden (bijlage A, blz. 19)

4. APPARATUURKOSTEN

nr	Omschrijving	Soort apparatuur computer of overig	Investeringsbedrag €	Afschrijving * 2010 €	Afschrijving * 2011 €	Afschrijving * 2012 €	Afschrijving * 2013 €	Afschrijving * 2014 €	**Totaal afschrijvingen projectperiode**
1				-	-	-	-	-	-
2									-
3									-
4									-
5									-
6									-
7									-
8									-
9									-
10									-
11									-
12									-
13									-
14									-
15									-
16									-
17									-
18									-
19									-
20									-
	**Totaal apparatuurkosten**		**-**	**-**	**-**	**-**	**-**	**-**	**-**

Ronde 2010 -2012 DoelmatigheidsOnderzoek

Aanvraagnummer: 80-82310-97-10061
Dr. Mw I. Draskovic
An RCT study of (cost) effectiveness of Dementia Care Mapping intervention in nursing home settings

5.a OVERIGE KOSTEN 2010

Omschrijving		Kosten
		-
		-
		-
		-
**Totaal overige kosten 2010**		**-**

5.b OVERIGE KOSTEN 2011

Omschrijving		Kosten
		-
		-
		-
		-
**Totaal overige kosten 2011**		**-**

5.c OVERIGE KOSTEN 2012

Omschrijving		Kosten
		-
		-
		-
		-
**Totaal overige kosten 2012**		**-**

5.d OVERIGE KOSTEN 2013

Omschrijving		Kosten
		-
		-
		-
**Totaal overige kosten 2013**		**-**

5.e OVERIGE KOSTEN 2014

Omschrijving		Kosten
		-
		-
		-
**Totaal overige kosten 2014**		**-**

Ronde 2010 -2012 DoelmatigheidsOnderzoek

Aanvraagnummer: 80-82310-97-10061
Dr. Mw I. Draskovic
An RCT study of (cost) effectiveness of Dementia Care Mapping intervention in nursing home settings

6.a BIJDRAGEN 2010

6.a.1	Eigen bijdrage instelling	-
6.a.2	Bijdragen derden:	-
		-
		-
		-
	**Totaal bijdragen 2010**	-

6.b BIJDRAGEN 2011

6.b.1.	Eigen bijdrage instelling	-
6.b.2	Bijdragen derden	-
		-
		-
		-
	**Totaal bijdragen 2011**	-

6.c BIJDRAGEN 2012

6.c.1.	Eigen bijdrage instelling	-
6.c.2	Bijdragen derden	-
		-
		-
		-
	**Totaal bijdragen 2012**	**-**

6.d BIJDRAGEN 2013

6.d.1.	Eigen bijdrage instelling	-
6.d.2	Bijdragen derden	-
		-
		-
	**Totaal bijdragen 2013**	**-**

6.d BIJDRAGEN 2014

6.e.1.	Eigen bijdrage instelling	-
6.e.2	Bijdragen derden	-
		-
		-
	**Totaal bijdragen 2014**	**-**

Flow chart DCM-NH intervention

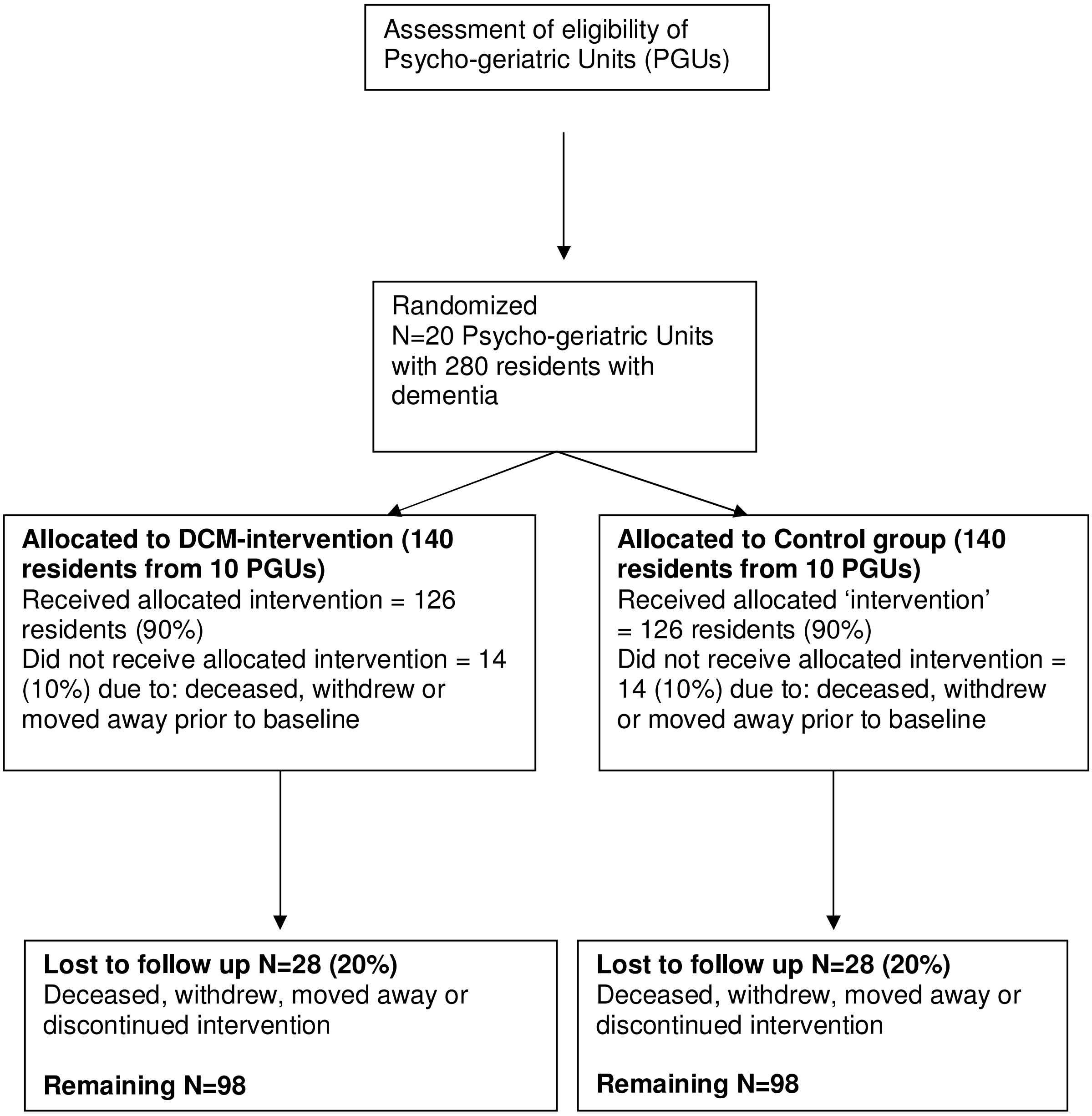

UMC St Radboud,
T.a.v. mw. dr. I. Draskovic
IQ healthcare
Postbus 9101, huispost 114
6500 HB Nijmegen

Geachte Mw. Draskovic

Ik heb uw projectvoorstel betreffende de DCM-studie goed gelezen. Ik heb contact gehad met het hoofd communicatie van V&VN, Monique Roedoe. Zij vindt het een goed idee om onze organisatie bij het onderzoek te betrekken. Met name omdat DCM veel verpleegkundige en verzorgende interventies betreft en het voor onze achterban een professionale ontwikkeling is.
Mochten jullie in de toekomst op zoek zijn naar instellingen, verzorgenden en/of verpleegkundigen die mee willen werken aan jullie onderzoek kun je ook gebruik maken van onze kanalen. Ook is er een mogelijkheid om een artikel voor te leggen aan de redactie van ons V&VN magazine.
Ik wens jullie veel succes met de projectaanvraag en ben uiteraard zeer benieuwd naar de voortgang.

Met vriendelijke groet,

Anna Zwanenburg
regiocoördinator V&VN
regio Midden-Oost Nederland

a.zwanenburg@venvn.nl

06-10905178

Verpleegkundigen & Verzorgenden Nederland
Churchilllaan 11, 3527 GV Utrecht
Postbus 8212, 3503 RE Utrecht
T (030) 291 90 50
F (030) 291 90 59

Samenvatting wijzigingen subsidieaanvraag

Projectnr. 80-82310-97-10061
Titel: 'An RCT study of (cost)effectiveness of Dementia Care Mapping intervention in nursing home settings'

Voorwaarden bij de voorgenomen besluit:

1. *"U dient de effectmaten nader te preciseren. De effectmaten afkomstig uit het voorstel van 11.94/23=0.52 lijken te optimistisch, gezien de in de wederhoor genoemde effectmaten van 0.31/0.32" Bij telefonische navraag werd met de contactpersoon mw. van Rossum afgesproken om de power analyse opnieuw uit te voeren en de sample size te berekenen op basis van de nieuwe gegevens uit de RCT-studie van Chenoweth et al (2009).*

   - Wijzigingen in de tekst projectvoorstel hoofdstuk Plan van aanpak, secties Sample size calculation en Time schedule:
     - **Pg. 11**- We hebben samen met onze methodoloog de *sampel size* opnieuw berekend. Aangezien de standaard deviatie berekend op basis van de gegevens uit de meest recente Australische RCT-studie kleiner was dat die uit de pilot studie en dankzij iets kleinere drop out percentages, zijn er ondanks verschillen in gemiddelden, geen wijzigingen in de grote van de steekproef opgetreden. De genoemde RCT studie is methodologisch verantwoord uitgevoerd waardoor de gegevens als betrouwbaar beschouwd kunnen worden.
     - **Pg. 14**- In verband met de verwachtingen t.o. van de drop out percentage hebben we onze tijdsplanning aangepast en gelijk aan die uit de Chenoweth et al. studie gemaakt.
     - Sammenvatting (**pg 3 en 4**) is aagepast conform de 2 punten

2. *"U wordt verzocht de begroting aan te passen. Onderstaande voorwaarden dient u in een nieuwe begroting op te nemen.*
   - *Personele kosten: De door u twee keer opgevoerde post projectbegeleiding dient terug gebracht te worden naar eenmaal projectbegeleiding (salarisschaal 11 met een maximale inzet 5% fte). De opgevoerde kosten van het secretariaat worden niet vergoed, aangezien dit overheadkosten betreffen."*

Wijzigingen personele kosten:

- **PG- 17, Bijlage -** De twee keer opgevoerde post projectbegeleiding is teruggebracht tot 1 keer en in plaats van 0.4 fte is het nu 0.2 fte. De huidige omvang van de post projectbegeleiding is gebaseerd op de complexiteit en omvang van de interventie. Naast de gebruikelijke werkzaamheden is het bv. nodig om bijeenkomsten (symposia) te organiseren met de mensen uit het veld, opleidingscomponent te organiseren en door te voeren en goede contacten op te bouwen en te onderhouden met de professionals om t.z.t. een bredere implementatie te waarborgen. Daarvoor is de ervaring en inzet van de senior onderzoeker onontbeerlijk.

- De kosten van het secretariaat zijn geschrapt. Echter, het zou de efficiëntie van het project ten goede komen als er in beperkte mate een projectgebonden secretariële ondersteuning mogelijk zou zijn.
- Wat de gevraagde wijzigingen in materiële kosten betreft, we hebben nu ten behoeve van het proefschrift precies de door u aangegeven bedrag van 1200,00 begroot.

Wij hopen hiermee aan alle door u gestelde voorwaarden te hebben voldaan en zien uw reactie met belangstelling tegemoet.

Met vriendelijke groet,
Mede namens de projectgroep,

Irena Draskovic
Projectleider en penvoerder
